# AIEE Active Nanoassemblies of Pyrazine Based Organic Photosensitizers as Efficient Metal-Free Supramolecular Photoredox Catalytic Systems

**DOI:** 10.1038/s41598-019-47588-5

**Published:** 2019-07-31

**Authors:** Shruti Dadwal, Harnimarta Deol, Manoj Kumar, Vandana Bhalla

**Affiliations:** Department of Chemistry, UGC Sponsored Centre for Advanced Studies-II Guru Nanak Dev University, Amritsar, 143005 Punjab India

**Keywords:** Self-assembly, Photocatalysis

## Abstract

Pyrazine derivatives **DIPY**, **TETPY** and **CNDIPY** have been designed and synthesized which form fluorescent supramolecular assemblies in mixed aqueous media due to their AIEE and ICT characteristics. Among all the derivatives, the assemblies of **TETPY** and **CNDIPY** show strong absorption in the visible region with high absorption coefficients, low HOMO-LUMO gap, and high photostability. Further, the supramolecular nanoassemblies of **TETPY** and **CNDIPY** show excellent potential to generate reactive oxygen species (ROS) under the visible light irradiation. Owing to their strong absorption in the visible region and ROS generation ability, the supramolecular nanoassemblies of **TETPY** and **CNDIPY** act as efficient photoredox catalytic systems for carrying out (a) oxidative amidation of aromatic aldehydes (b) hydroxylation of boronic acid and (c) oxidative homocoupling of benzylamines under mild conditions such as aqueous media, aerial environment, and natural sunlight as a source of irradiation. All the mechanistic investigations suggest the participation of *in-situ* generated ROS in the organic transformations upon light irradiation.

## Introduction

Photoredox catalysis is a promising ‘green’ economic approach to speed up oxidative organic reactions by utilizing sunlight as a source of energy^1^. The activity of a photoredox catalyst is determined by its potential to generate reactive oxygen species (ROS) upon light irradiation which in turn is dependent upon the intersystem crossing (ISC) of generated excitons. The lifetime of the triplet state of the materials gives an overview of the extent of the intersystem crossing of excitons under the visible light irradiation^2,3^. Most of the conventional photoredox catalysts are limited to off-the-shelf compounds such as organometallic complexes (Ru(bpy)_3_Cl_2_, Ir(bpy)_3_Cl_2_) and commercially available organic dyes (methylene blue, Eosin Y, Rhodamine, Rose Bengal, etc.) in which precious and toxic metals are incorporated to ensure high intersystem crossing^4–7^. The organometallic complexes have long/tedious synthetic routes. Additionally, most of these materials have a quite low molar extinction coefficients as a result of which their high loading is needed in the reactions to achieve high yield of the target compounds. On the other hand, most of the commercially available organic dyes have low photostability and it is also difficult to alter their molecular structure to modulate their photophysical properties. Over the past few years, the pursuit of researchers to make organic transformations more ‘green’ resulted in the development of new organic photosensitizers (such as BODIPY derivatives and carbon-based derivatives) having strong absorption in the visible region and long-lived triplet states^8–13^. Though these photosensitizers could overcome some of the limitations associated with off-the-shelf complexes/dyes such as low photostability and requirement of high catalytic loading, however, presence of toxic heavy atoms and their multi-step synthesis (especially in carbon-based materials) which actually undermines the overall goal of an alternative ‘green’ catalytic system.

**Figure 1:**
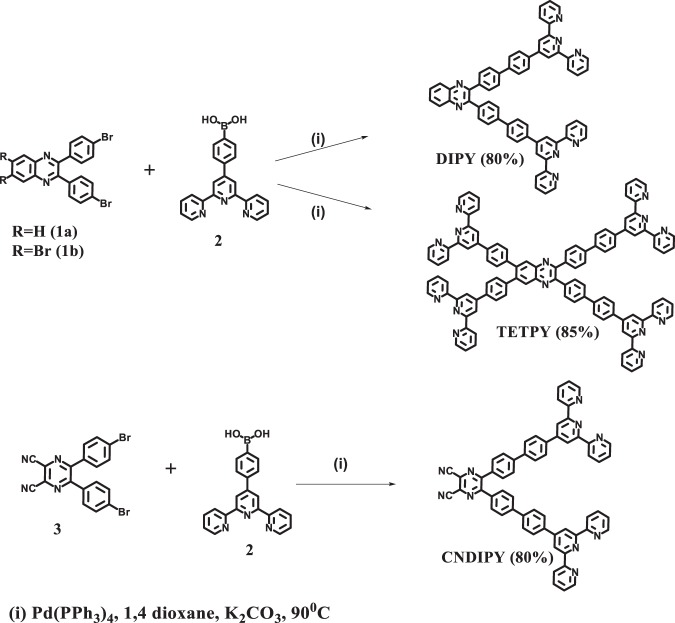
Synthesis of pyrazine-based donor-acceptor systems **DIPY**, **TETPY**, and **CNDIPY**. Reaction Conditions: (i) Pd(PPh_3_)_4_, K_2_CO_3_(aq.), 1,4- dioxane, reflux at 90° C under N_2_ atmosphere.

Very recently, supramolecular assemblies of perylene bisimide (PBI) derivatives having the potential to generate reactive oxygen species have been reported as efficient photosensitizers for degradation of dyes and photodynamic therapy under visible light irradiation^14^. This strategy is convenient and inspired us to develop supramolecular assemblies as photoredox catalytic systems for oxidative organic transformations. Since aggregation-induced emission enhancement (AIEE) materials are known for their high photostability^15^, hence, we planned to synthesize donor-acceptor systems like **DIPY**, **TETPY**, and **CNDIPY** (Fig. 1) using AIEE active pyrazine (Py) scaffold as acceptor moiety and terpyridine (Tpy) as donor group(s). Besides the high photostability, we envisaged that terpyridine and pyrazine based donor-acceptor systems will exhibit strong absorption in the visible region due to intramolecular charge transfer (ICT) transition. Amazingly, all the pyrazine derivatives show ICT and AIEE characteristics and the assemblies of **TETPY** and **CNDIPY** were found to exhibit strong absorption in the visible region with high molar extinction coefficients and low HOMO-LUMO gap (E_g_) as compared to the assemblies of **DIPY** derivative. All the derivatives show remarkable photostabilities and high potential to generate superoxide radicals/singlet oxygen in the aggregated state when exposed to visible light radiation in comparison to their isolated forms. Interestingly, the as-prepared supramolecular assemblies of pyrazine derivatives **TETPY** and **CNDIPY** exhibit high photocatalytic activity in; (a) oxidative coupling of benzylamines (b) additive/base free oxidative amidation of aldehydes and (c) hydroxylation of boronic acid in mixed aqueous media under mild conditions using natural sunlight as the source of irradiation. The present photoredox catalytic systems are found to be better than the other catalytic systems reported in the literature (Comparison Tables S1–S3 in the Supplementary). Most importantly, in all the reactions, low catalytic loading (0.1 mol%) of the photoredox system was used.

To best of our knowledge, this is the first report which demonstrates the development of AIEE-ICT active ‘metal/heavy atom’ free supramolecular nanoassemblies of pyrazine based photosensitizers as efficient photoredox catalytic systems for carrying out oxidative organic transformations using natural sunlight as the source of irradiation.

## Results and Discussion

### Synthesis of the pyrazine derivatives

Suzuki Miyaura coupling between dibromo pyrazine/tetrabromo pyrazine derivative (**1a/1b**)^16^ and 4-(2,2′,6′,2″-terpyridine-4′-yl) phenyl boronic acid (**2**)^17^ in dioxane furnished **DIPY**/**TETPY** derivative in 80%/85% yield (Fig. 1). The ^1^H NMR spectrum of **DIPY**/**TETPY** in CDCl_3_ shows a characteristic singlet at 8.80/8.80 ppm corresponding to Tpy aromatic protons and characteristic doublet/multiplet at 8.23/8.36-8.33 ppm corresponding to Py aromatic protons. The parent ion peak corresponding to **DIPY** appears at 897.365 in ESI-MS spectrum. The peak corresponding to **TETPY** appears at 1512.518 [M + H]^+^ in ESI-MS spectrum. These spectroscopic data corroborate the structure **DIPY**/**TETPY** for this compound (Figs S31–S36 in the Supplementary).

The **CNDIPY** was synthesized in 80% yield by Suzuki Miyaura coupling between bromo derivative of dicyanopyrazine (**3**)^18^ and 4-(2,2′,6′,2″-terpyridine-4′-yl) phenyl boronic acid (**2**)^17^. The ^1^H NMR spectrum of **CNDIPY** in CDCl_3_ shows two characteristic singlets at 8.84 and 8.80 ppm corresponding to Tpy aromatic protons. In the ESI-MS, a peak corresponding to **CNDIPY** appears at 936.2809 [M + K]^+^. These spectroscopic data corroborate the structure **CNDIPY** for this compound (Figs S37–S39 in the Supplementary).

### Photophysical and electrochemical behavior of pyrazine derivatives

**Figure 2:**
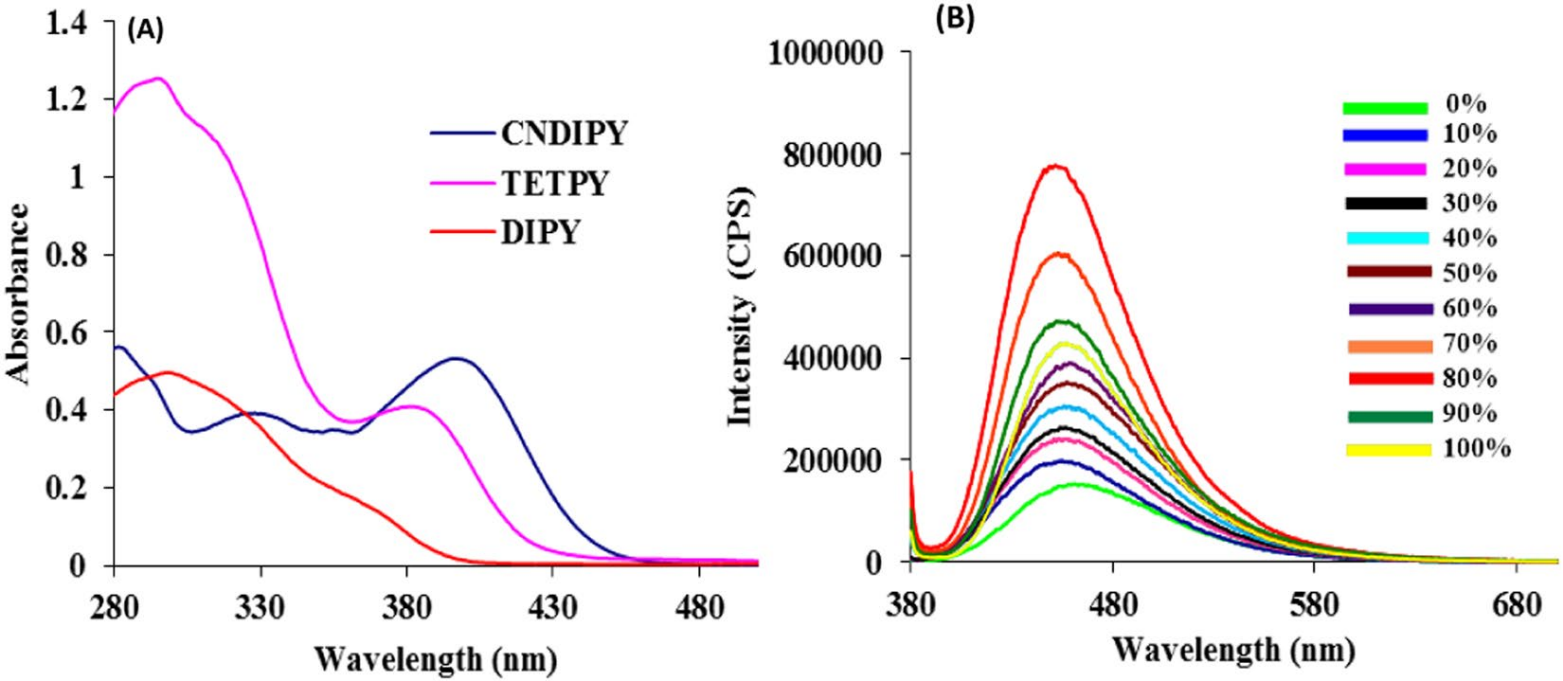
(**A**) UV-vis spectra of **DIPY**, **TETPY**, and **CNDIPY** derivatives in DMSO/H_2_O. (**B**) The fluorescence spectra of **CNDIPY** in different fractions of DMSO and water at λ_ex_ = 370 nm.

We examined the photophysical behavior of all the synthesized pyrazine derivatives by UV-vis and fluorescence studies. Two absorption bands appear in the UV- vis spectra of all the derivatives. The absorption band in the range of 290–330 nm is attributed to the π -π* transitions of pyrazine core^19^ and the second absorption band observed at longer wavelength (370–400 nm) is attributed to the intramolecular charge transfer (ICT) transitions (Fig. 2A and Table 1). Both **TETPY** and **CNDIPY** derivatives exhibit relatively strong absorption in the visible region. Further, to confirm the ICT characteristics of these derivatives, we carried out the fluorescence studies in different solvents. Interestingly, all the derivatives show positive solvatochromic behavior (Figs S1–S3 in the Supplementary) which confirms their ICT characteristics. To examine the aggregation induced enhanced emission (AIEE) characteristics of the synthesized compounds, we carried out the fluorescence studies of pyrazine derivatives in different fractions of DMSO/H_2_O solvent mixtures. Upon gradual addition of water upto 80% to DMSO solution of **CNDIPY**, a five-fold enhancement in the intensity of the emission band at 460 nm is observed (Fig. 2B). Likewise, derivative **TETPY** (2.1 fold) and **DIPY** (4.9 fold) show emission enhancement upon addition of water fraction to their DMSO solution (Figs S4,S5 in Supplementary). The viscosity dependent fluorescence studies of **DIPY**, **TETPY** and **CNDIPY** derivatives in DMSO/glycerol solvent mixture show the enhancement in the emission intensity which confirms that restriction to intramolecular rotation is the reason behind the AIEE phenomenon (Figs S6–S8 in the Supplementary). After examining the AIEE characteristics of the pyrazine derivatives, their photostability was also checked and for this, we exposed the DMSO: H_2_O solutions of all the derivatives to continuous visible irradiation for 36 h and monitored the change in their absorption behavior using UV-vis spectroscopy. To our pleasure, no significant change in the absorption behavior was observed in case of all the derivatives even after 36 h of continuous exposure.

**Table 1:** Photophysical and electrochemical data for pyrazine derivatives.

Pyrazine derivatives	λ_abs_ (nm)	λ_em_ (nm)	Molar extinction coefficient (M^−1^ cm^−1^)	E_1/2red_^a^ (V)	E_g_ (eV)
CNDIPY	330/400	460	50,000	−1.7	2.67
TETPY	292/390	450	40,000	−1.76	2.76
DIPY	296/370	440	20,000	−1.64	3.10

E_1/2_red^a^ are half-wave reduction potential measured in ACN versus Ag/AgCl. Eg (HOMO-LUMO gap) calculated from Cyclic Voltammogram.

These results highlight the remarkable photostability of supramolecular assemblies of all the pyrazine derivatives (Figs S9–S11 in the Supplementary)

We further examined the morphology of **TETPY** and **CNDIPY** derivatives by transmission electron microscopy (TEM). The TEM images of **TETPY** derivative show the presence of spherical shaped assemblies having the size in a range of 50–100 nm while irregular assemblies with size in the range of 50–100 nm were observed in case of **CNDIPY** derivative. (Fig. S12A,B in Supplementary). The size of nanoassemblies was also determined by dynamic light scattering (DLS) experiments which corroborated very well with TEM studies (Fig. S12C in Supplementary). The energy dispersive X-ray (EDX) spectrum of **TETPY** derivative shows the absence of any metallic content (Fig. S13 in Supplementary).

Next, we examined the electrochemical behavior of all the pyrazine derivatives to determine their HOMO-LUMO energy gaps (E_g_)^18^. We observed the tuning of the energy gaps with the change in the acceptor strength (**CNDIPY**) and donor strength/extent of conjugation (**TETPY**). From the cyclic voltammetric studies, **CNDIPY** showed the smallest HOMO–LUMO gap which may be attributed to its strong push-pull behavior (Table 1) (Fig. S14 in the Supplementary). The low E_g_ values of these materials emphasize their potential to generate radicals through a single electron transfer (SET) mechanism.

The HOMO–LUMO gap was also evaluated using density functional theory (DFT) calculations and these results very well corroborated the experimental studies (Figs S15–S17 in the Supplementary).

**Figure 3:**
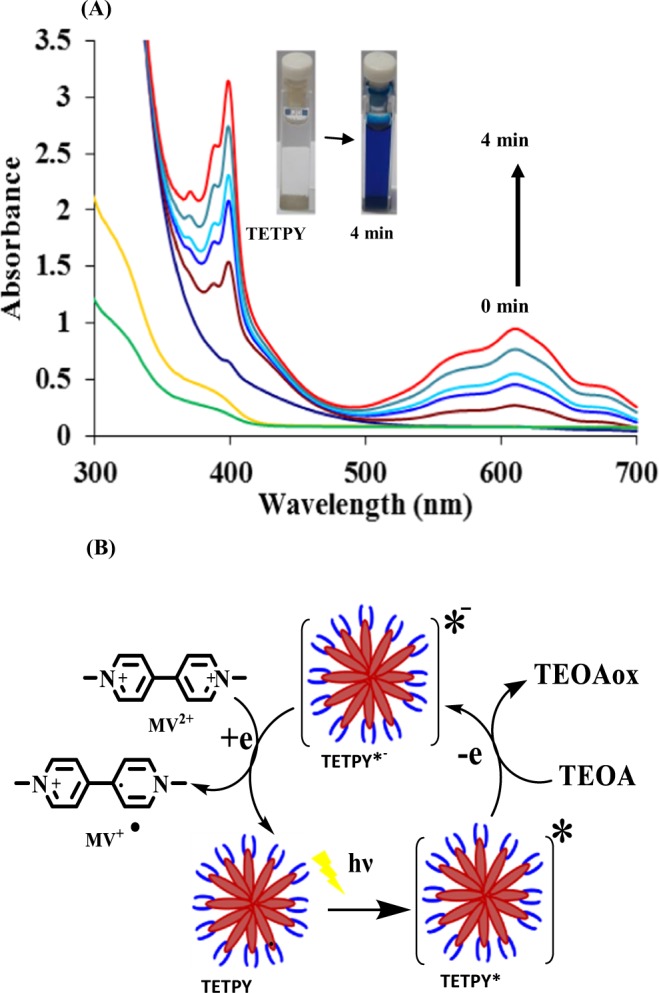
(**A**) Absorption spectral changes of derivative **TETPY** (0.02 mM) in presence of MV^2+^ (0.2 mM) and TEOA (50 mM) in DMSO: H_2_O under day-light and inert atmosphere. (**B**) Pyrazine derivative **TETPY** acts as photosensitizer and transports the electron to a sacrificial donor to acceptor under visible light irradiation and inert atmosphere.

Next, we evaluated the role of pyrazine assemblies to act as photosensitizers by examining their potential in the transportation of electrons from donor to acceptor moiety. For carrying out these studies, methyl viologen (MV^2+^) and triethanolamine (TEOA) were chosen as the electron acceptor and sacrificial donor materials, respectively. Upon addition of TEOA (50 mM) to the solution of supramolecular assemblies of **TETPY** in DMSO: H_2_O (1:1), a 30% decrease in the intensity of the absorption band was observed. This observation suggests the existence of strong interactions between a sacrificial donor and supramolecular assemblies of **TETPY**. Thereafter, we added MV^2+^ (0.2 mM) to the solution and exposed it to the day-light under an inert atmosphere. The color of the solution changed from colorless to dark blue after 4 minutes. The absorption spectrum of the solution in the presence of methyl viologen showed the formation of two new bands at 395 and 603 nm, respectively. These two bands are due to the presence of reduced cationic species ${{\rm{MV}}}^{\cdot +}$ of methyl viologen (Fig. 3A). The intensity of both bands gradually increased upon exposure to day-light. These observations led us to propose that assemblies of **TETPY** are reductively quenched by a sacrificial donor to generate radical anion (TETPY^*−^) which in turn transports electron to MV^2+^ and reduce it (Fig. 3B)^20^. The blue colored radical cation persisted for 2 h and was slowly reoxidized upon exposure to aerial conditions. Similarly, supramolecular assemblies of **CNDIPY** also showed the color change within five minutes from colorless to blue (Fig. S18 in the Supplementary). These results demonstrate the potential of the supramolecular assemblies of **TETPY** and **CNDIPY** to act as efficient photosensitizers. However, in case of supramolecular assemblies of **DIPY**, the color change was slow (15 min) which may be attributed to their weak absorption in the visible region (Fig. S19 in the Supplementary).

Thereafter, we evaluated the potential of the synthesized materials to generate reactive oxygen species (ROS) and for this we employed 9,10-anthracenediyl-bis(methylene) dimalonic acid (ABDA) as a singlet oxygen scavenger. The ability of ABDA to get consumed upon reaction with *in-situ* released singlet oxygen by photosensitizer upon irradiation is well documented^21–24^. The absorption range of ABDA is very close to that of assemblies of pyrazine derivatives. To rule out the possibility of self-quenching of absorption of ABDA due to its simultaneous excitation upon exposure to radiation, we performed a blank experiment by exposing the solution of ABDA to light radiation for 20 minutes. No significant change in the absorption of ABDA was observed^25^ (Fig. S20 in the Supplementary). These results encouraged us to use ABDA as an oxygen scavenger to determine the ROS generation potential of the synthesized materials. The solution of **CNDIPY** (5 µM) in DMSO: H_2_O (1:1) containing ABDA was aerated for 5 minutes before its exposure to radiation. After aeration, the solution was exposed to light radiation (λ = 400 nm) for 2 minutes and its absorption was monitored using UV-vis spectroscopy.

**Figure 4:**
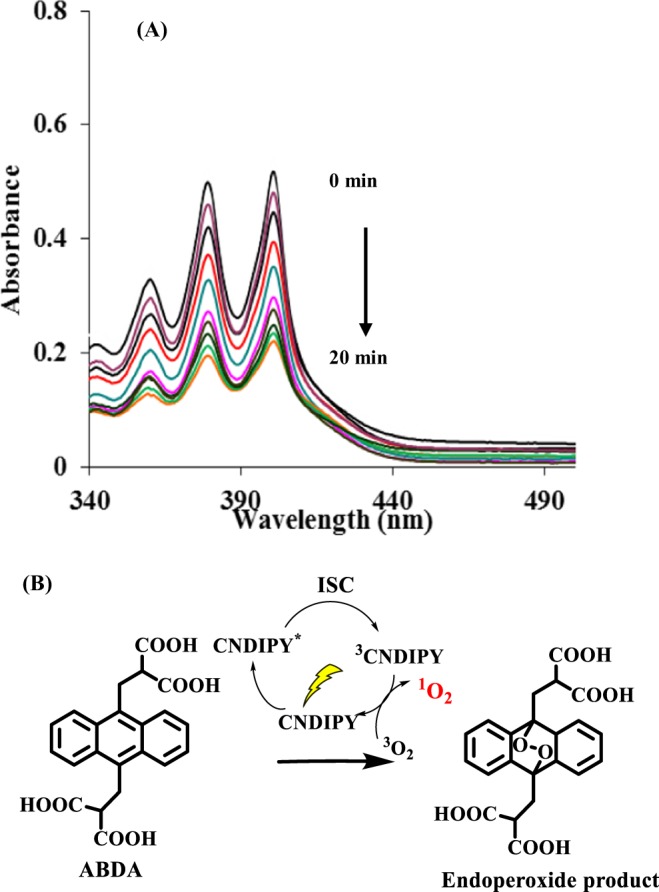
(**A**) UV-vis spectra of ABDA in presence of nanoassemblies of **CNDIPY** under irradiation of visible light. (**B**) The chemical reaction of ABDA with *in-situ* generated singlet oxygen (^1^O_2_) by **CNDIPY** upon photoirradiation.

This procedure was repeated several times to achieve saturation (20 minutes). The absorption studies show a 60% decrease in absorption intensity of ABDA upon total exposure to radiation for 20 minutes (Fig. 4A,B). However, a 35% consumption of ABDA was observed when the experiment was carried out in its isolated state (DMSO solution of **CNDIPY**) (Fig. S21 in the Supplementary). These studies show that AIEE active supramolecular nanoassemblies of **CNDIPY** have more photosensitizing ability in aggregated state than in the isolated state.

**Figure 5:**
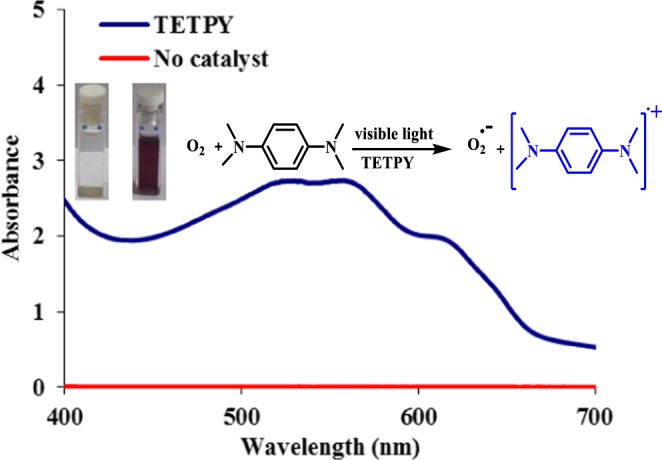
UV−vis absorption spectra and photographs of the cationic radical species of N,N,N′,N′-tetramethyl-p-phenylenediamine generated by assemblies of **TETPY** (5 µM) in the presence of visible light and oxygen.

The nanoassemblies of **TETPY** [5 µM, in DMSO: H_2_O (1:1)] consumed 50% of the total ABDA upon exposure to light radiation (λ = 400 nm) for 20 minutes and only 25% of total ABDA was consumed in the isolated form of **TETPY** derivative (Figs S22 and S23 in the Supplementary). However, in the presence of **DIPY** nanoassemblies, a 20% decrease in the absorption intensity of ABDA was observed after total exposure of 20 minutes (Fig. S24 in the Supplementary). Overall, the fast rate of consumption of ABDA in the presence of supramolecular assemblies of **CNDIPY** and **TETPY** show their good potential towards the generation of singlet oxygen. Further, we carried out the cyclic voltammetric studies of all the synthesized derivatives to determine their potential to reduce the molecular oxygen to superoxide radical. Both **TETPY** and **CNDIPY** derivatives show sufficient reduction potential (Table 1) to reduce molecular oxygen to generate superoxide radical (O_2_^−^) species (Fig. S14 in the Supplementary). These results prove that nanoassemblies of pyrazine derivatives have the ability to generate superoxide radicals under visible light irradiation. We also confirmed the generation of superoxide radicals by supramolecular assemblies of pyrazine derivatives upon exposure to visible light by using *N*,*N*,*N*′,*N*′-tetramethyl-*p*-phenylenediamine (TMPD) as indicator^26–28^. TMPD was added to the solution of supramolecular assemblies of **TETPY** [5 µM, in DMSO:H_2_O (1:1)] and the solution was kept for 5 minutes under the irradiation of the visible light. Initially, the solution was colorless then it changed to blue after exposure of 5 minutes. The absorption spectrum of the solution show the formation of new absorption bands at 564 and 613 nm which may be attributed to the oxidation of TMPD and generation of cationic radical of TMPD due to the release of superoxide radical (Fig. 5).

It took 10 minutes to observe the color change in case of assemblies of **CNDIPY** (Fig. S25 in the Supplementary). The quick response (5 minutes) in case of assemblies of **TETPY** suggests their relatively stronger electron transport ability and stronger superoxide radical generation ability. In the case of supramolecular assemblies of **DIPY**, the color change is observed after 20 minutes of exposure (Fig. S26 in the Supplementary)

## Photocatalytic activity of the supramolecular assemblies of pyrazine derivatives in the presence of natural sunlight

### Oxidative homocoupling of benzylamine

**Figure 6:**
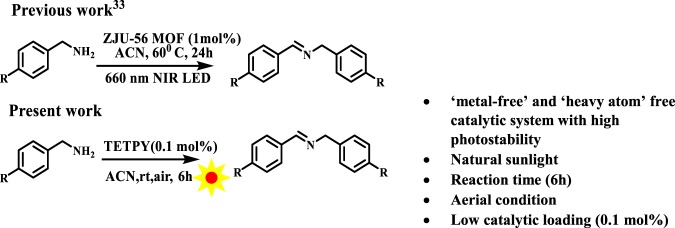
Oxidative homocoupling of benzylamines.

Supramolecular nanoassemblies of **TETPY** and **CNDIPY** possess all the desirable characteristics of photoredox catalytic systems due to their excellent photostability, low HOMO-LUMO gap, high absorption coefficient in the visible region, and good ROS generation abilities. Keeping this in view, we planned to examine the oxidative coupling of benzylamines using supramolecular assemblies of pyrazine derivatives as photoredox catalytic systems. The selective oxidation of amines to the corresponding imines is one of the most significant functional group conversions since imines and their derivatives are important building blocks for the synthesis of biomolecules, pharmaceuticals and valuable chemicals^29,30^. In literature, a variety of photosensitizers based on commercially available organic dyes, BODIPY, MOF, and polymers have been used for the oxidative coupling of the benzylamines^29–32^. The utilization of heavy atoms in organic photosensitizers, costly metal-based complexes in MOFs, tedious synthetic routes of COFs, and polymers restrict their large scale photocatalytic applications. Moreover, in most of the cases blue LED is utilized as the source of irradiation. Recently, zinc-based MOFs have been utilized for carrying out homocoupling of benzylamines using NIR LED^33,34^. Though the utilization of NIR light is advantageous, however, the heating (60 °C) is needed for the longer period (24 h) to get good yields of the product which actually decreases the ‘green’ benefits of the approach (Fig. 6).

**Figure 7:**
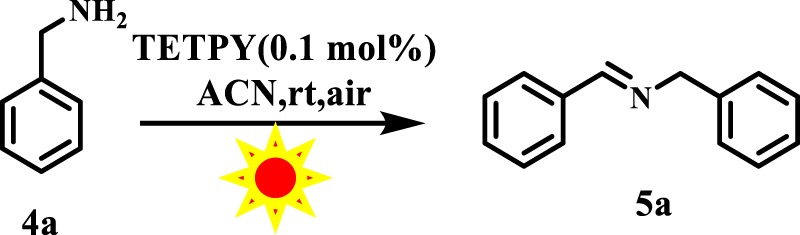
The model reaction.

In the present investigation, we carried out the model reaction of benzylamine in acetonitrile (ACN) under tungsten bulb irradiation and aerial conditions using assemblies of **TETPY** as photoredox catalytic systems (Fig. 7). To our pleasure, the reaction proceeded well and the desired product was obtained in 82% yield (Table 2, entry 1). When the model reaction was carried out under natural sunlight, the desired product was obtained in 85% yield (Table 2, entry 2).

**Table 2:** Optimization of reaction conditions.

Entry	Photocatalyst (Nanoassemblies)	Solvent	Time	Yield%
1	TETPY	ACN	6 h	82
2^a^	TETPY	ACN	6 h	85
3^b^	TETPY	ACN	6 h	68
4	CNDIPY	ACN	6 h	85
5^b^	CNDIPY	ACN	6 h	65
6	DIPY	ACN	6 h	45
7	TETPY	CH_2_Cl_2_	6 h	59
8	TETPY	ACN:H_2_O(1:1)	6 h	30
9	TETPY	DMSO	6 h	30
10	TETPY	THF	6 h	45
11^c^	—	ACN	6 h	NR
12^d^	TETPY	ACN	6 h	NR
13^e^	TETPY	ACN	6 h	NR

^a^natural sunlight, ^b^Isolated form, ^c^No photocatalyst, ^d^No light, ^e^Inert atmosphere, NR No reaction.

These results indicate that the assemblies of **TETPY** act more efficiently under irradiation of natural sunlight. Hence, we utilized natural sunlight as a source of irradiation for carrying out the next organic transformations. We repeated the model reaction using **TETPY** in its isolated form as photocatalysts, the desired product was obtained in only 68% yield (Table 2, entry 3). The model reaction was also performed with **CNDIPY** in its aggregated and isolated form and the target products were obtained in 85% and 65% yields, respectively (Table 2, entries 4, 5). These results show that supramolecular assemblies of pyrazine derivatives have more photocatalytic efficiency than their monomeric analogs. The desired product was obtained in 45% yield in case of assemblies of **DIPY** (Table 2, entry 6). The low catalytic efficiency in case of assemblies of **DIPY** may be attributed to their low absorption in the visible region. Further, we examined the effect of different solvents in oxidative homocoupling reaction of benzylamines by switching the solvents from DCM, ACN: H_2_O, DMSO to THF and in all the cases the desired product was furnished in lower yields (Table 2, entries 7–10).

We performed a series of experiments to achieve the optimized conditions and all the control experiments (Table 2, entries 11–13) show that presence of assemblies of pyrazine derivative as photocatalysts, natural sunlight, aerial conditions, and ACN as solvent are essential for achieving the good yields.

**Figure 8:**
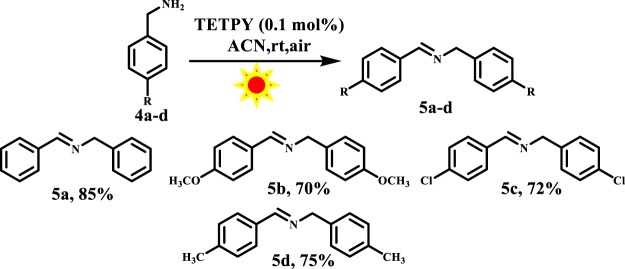
Oxidative coupling of substituted benzylamines using supramolecular assemblies of **TETPY** as photocatalysts under natural sunlight and aerial conditions.

With optimized conditions in hand, we examined the catalytic activity of assemblies of **TETPY** toward various substituted benzylamines and in all the cases the desired product was obtained in good yields (Fig. 8).

To get insight into the mechanistic pathways, we carried out the model reaction using assemblies of **TETPY** as photocatalysts in the presence of singlet oxygen scavengers such as sodium azide^35^ and DABCO^36^, the desired product was obtained in 30% and 28% yields, respectively. The model reaction was also carried out with superoxide scavenger such as 1,4 benzoquinone^37^ using assemblies of **TETPY** as photocatalysts and the yield of the desired product decreased to 38%. (Fig. S27 in the Supplementary).

**Figure 9:**
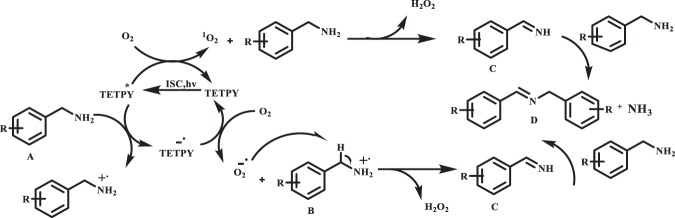
Proposed mechanism of oxidative coupling of benzylamines using supramolecular assemblies of **TETPY** as photocatalysts under natural sunlight and aerial conditions.

On the basis of these observations, we propose that singlet oxygen and superoxide radicals generated by assemblies of pyrazine derivative upon photoexcitation oxidized the benzylamine (A) to the imine derivative (C) with the release of H_2_O_2_. The imine derivative (C) reacted with another molecule of benzylamine to generate the desired product (D) (Fig. 9)^30^.

To validate the assumption of the release of H_2_O_2_ as by-product, we checked the *in-situ* generation of H_2_O_2_ by adding the N,N-diethyl-1,4-phenylenediammonium sulphate (DPD) and Horseradish peroxide (POD) in the reaction mixture.

**Figure 10:**
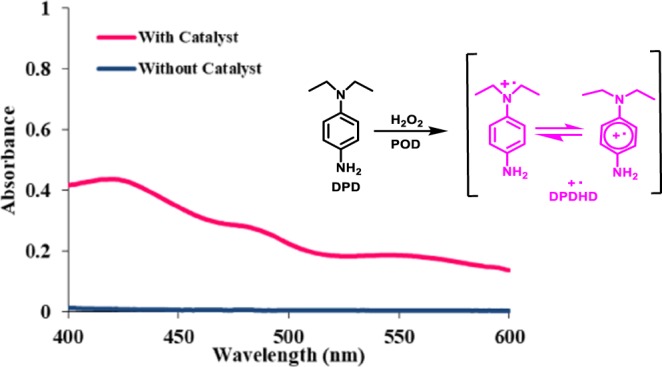
UV- vis absorption spectra of the benzylamine reaction system with or without catalyst after the addition of DPD and POD.

We envisaged that POD will catalyze the oxidation of DPD in presence of oxidizing agent *i*.*e* H_2_O_2_ and the chemical change will be clearly visible to naked eye^32,38^. Amazingly, the color of the reaction mixture turned pink which is attributed to the formation of DPD radical cation. The whole experiment was monitored by UV-vis studies which supports the above assumption (Fig. 10).

### Oxidative amidation of aromatic aldehydes

**Figure 11:**
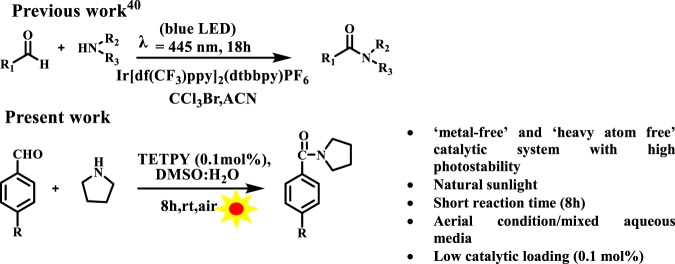
Oxidative amidation of aromatic aldehydes.

**Figure 12:**
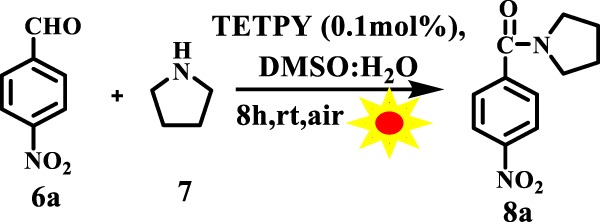
The model reaction.

The ability of supramolecular assemblies of **TETPY** and **CNDIPY** to efficiently catalyze the oxidative coupling of benzylamines motivated us to study the oxidative amidation of aromatic aldehydes using these assemblies as photoredox catalytic systems. The amide bond is a structural backbone of proteins and peptides and is also predominant in medicinally important compounds, natural products and polymers^39^. In literature, organometallic complexes, organic dyes, and triplet photosensitizers have been utilized for carrying out oxidative amidation of aromatic aldehydes using organic solvents, blue LED and in presence of additives and base^40–43^. Recently, light-mediated cross dehydrogenating coupling of aldehydes using iridium-based photocatalysts in presence of BrCCl_3_ as an additive has been reported (Fig. 11)^40^. In the present case, we chose reaction between 4-nitrobenzaldehyde and pyrrolidine using DMSO: H_2_O (1:1) as the model system under natural sunlight and aerial conditions. The model reaction catalyzed by assemblies of **TETPY** was monitored under the irradiation of natural sunlight and aerial conditions (Fig. 12). The desired product was obtained in 90% yield using nanoassemblies of **TETPY** after 8 h (Table 3, entry 1) in the absence of any additives and base. Further, we utilized the nanoassemblies of **CNDIPY** as photocatalysts under the optimized reaction conditions and the product was obtained in 85% yield (Table 3, entry 2).

**Table 3:** Optimization of reaction conditions.

Entry	Photocatalyst (Nanoassemblies)	Additive	Solvent	Time	Yield (%)
1	TETPY	—	DMSO:H_2_O (1:1)	8 h	90
2	CNDIPY	—	DMSO:H_2_O(1:1)	8 h	85
3	DIPY	—	DMSO:H_2_O(1:1)	8 h	45
4	TETPY	—	DMSO	8 h	85
5	TETPY	—	DMF	8 h	17
6	TETPY	—	Dioxane	8 h	22
7	TETPY	—	THF	8 h	54
8	TETPY	—	ACN	8 h	70
9	TETPY	—	Water	8 h	32
10	TETPY	K_2_CO_3_ as a base	DMSO:H_2_O(1:1)	8 h	90
11^a^	—	—	DMSO:H_2_O(1:1)	8 h	8
12^b^	TETPY	—	DMSO:H_2_O(1:1)	8 h	N.R
13^c^	TETPY	—	DMSO:H_2_O(1:1)	8 h	Traces

^a^No Photocatalyst, ^b^No light, ^c^Inert Atmosphere.

When the model reaction was repeated in the presence of assemblies of **DIPY** as photocatalysts, the desired product was obtained in 45% yield (Table 3, entry 3). We studied the effect of different solvents such as DMSO, DMF, dioxane, THF, ACN, water, and DMSO: H_2_O (Table 3, entries 4–9) using assemblies of **TETPY**. The maximum yield was obtained in case of DMSO: H_2_O as the solvent system and low yield was obtained in H_2_O. We believe that low solubility of reactants, as well as a catalyst in H_2_O, is the reason behind the low yield of the target product. We chose DMSO: H_2_O as the solvent system to carry out further organic transformations. We also examined the effect of presence/absence of the base in the oxidative amidation of aromatic aldehydes under natural sunlight irradiation (Table 3, entry 10). The presence or absence of base did not affect the rate and yield of reaction. The reaction was also carried out in the absence of **TETPY** and only 8% of the desired product was obtained (Table 3, entry 11). However, in the absence of light, the formation of desired product was not observed (Table 3, entry 12). The model reaction was also studied in an inert atmosphere and the desired product was obtained only in traces (Table 3, entry 13). These observations led us to conclude that the presence of photocatalyst, light and aerial conditions are the prerequisite to carry out the oxidative amidation of the aromatic aldehydes.

**Figure 13:**
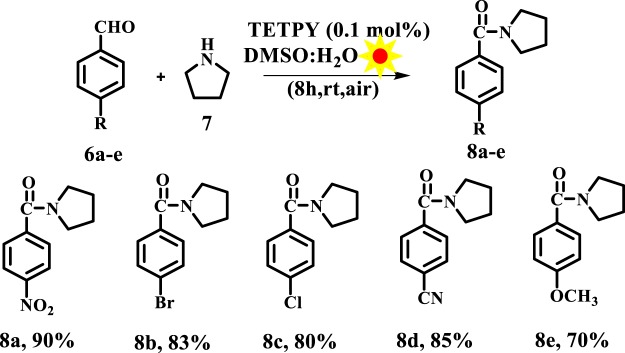
Oxidation amidation of substituted aromatic aldehydes with pyrrolidine using supramolecular assemblies of **TETPY** as photocatalysts under natural sunlight and aerial conditions.

Next, we examined the catalytic efficiency of supramolecular assemblies of **TETPY** using different aromatic aldehyde. Both aromatic aldehydes bearing electron withdrawing and electron donating groups furnished the desired products in excellent yields (Fig. 13).

### Mechanistic studies

To gain insight into the mechanism of amidation, several control experiments were performed. The model reaction in the presence of singlet oxygen scavengers such as sodium azide^35^ and DABCO^36^ using **TETPY** as photocatalysts under optimized conditions furnished the desired products in 40% and 38% yields respectively. Further, the model reaction in the presence of 1,4-benzoquinone^37^ (superoxide quencher) furnished the desired product in only 38% yield (Fig. S28 in the Supplementary). These results reveal that the reaction proceeds through the intermediary of both singlet oxygen and superoxide radicals. We also checked the effect of the H_2_O_2_ on the reaction kinetics by adding H_2_O_2_ to the reaction mixture. The yield of the desired product under optimized conditions increased to 95% in the presence of H_2_O_2._

These control experiments indicate that during reaction conditions, H_2_O_2_ is generated and *in-situ* generated H_2_O_2_ is promoting the reaction in the forward direction. Further, *in-situ* generation of H_2_O_2_ was also supported by POD catalyzed the oxidation of DPD (Fig. S29 in the Supplementary)^38^.

**Figure 14:**
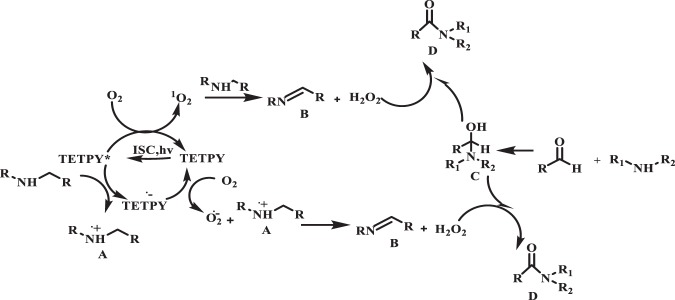
Proposed mechanism for oxidative amidation of aromatic aldehydes using supramolecular assemblies of **TETPY** under natural sunlight and aerial conditions.

On the basis of these results, we propose the mechanism in which the TETPY on photoexcitation and ISC goes to triplet state TETPY*. This excited TETPY* transfers energy to the dioxygen to generate singlet oxygen (^1^O_2_). This ^1^O_2_ reacts with amine to give intermediate (B) with the generation of H_2_O_2_. On the other hand, the amine transfers the electron to TETPY* in its triplet excited state to generate TETPY^−^. which further transfers electron to dioxygen to give superoxide radical. This superoxide radical reacts with radical cation (A) to generate intermediate (B) with the generation of H_2_O_2._ The *in-situ* generated H_2_O_2_ acts as an oxidant and is responsible for the oxidative amidation of aromatic aldehyde under the natural sunlight irradiation to give the desired product (D) through the formation of (C) (Fig. 14)^44^.

### Oxidative hydroxylation of boronic acids

**Figure 15:**
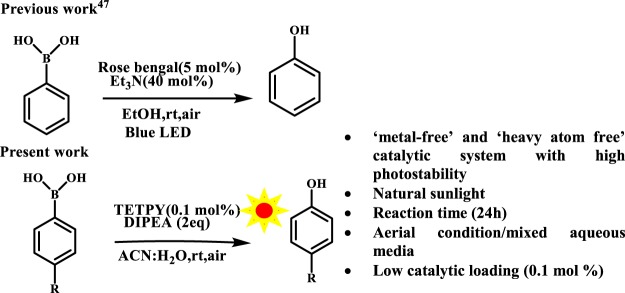
Oxidative hydroxylation of boronic acid.

Next, we explored the catalytic activity of assemblies of **TETPY** and **CNDIPY** in the hydroxylation of aryl boronic acids for the preparation of phenols. Development of new synthetic methods for preparation of phenol is important as phenol is a ubiquitous structural unit found in a number of natural products and pharmaceuticals^45,46^. Under the category of ‘metal-free’ catalytic system, Rose Bengal has been utilized for carrying out hydroxylation of boronic acid under blue light irradiation (Fig. 15), however the reaction conditions need 5 mol% of the catalysts along with 40 mol% of the base and toxicity of organic dye restricts their real-time application^47^.

**Figure 16:**
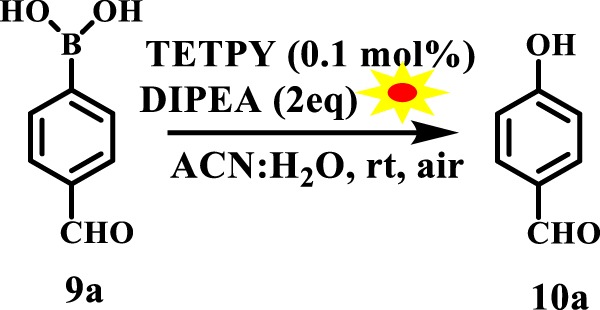
The model reaction.

To overcome these problems, we planned to carry out the hydroxylation of boronic acids using supramolecular assemblies of **TETPY** and **CNDIPY** as photocatalysts. We chose 4-formyl phenylboronic acid as reactant, N,N-diisopropylethylamine (DIPEA) as electron donor and supramolecular assemblies of **TETPY** as photocatalysts (Fig. 16) to furnish the desired product in 88% yield using ACN: H_2_O as a solvent system (Table 4, entry 1). The model reaction was also carried out with assemblies of **CNDIPY** as photocatalysts to furnish the product in 80% yield (Table 4, entry 2). Further, the reaction conditions were screened using different solvents such as DMSO, DMSO: H_2_O, ACN, H_2_O, DMF, MeOH and EtOH (Table 4, entries 3–9). The highest yield was obtained in case of ACN: H_2_O as solvent system. We chose ACN: H_2_O as reaction media and supramolecular assemblies of **TETPY** as photocatalyst for carrying out other reactions.

**Table 4:** Optimization of reaction conditions.

Entry	Photocatalyst (Nanoassemblies)	Solvent	Time	Yield (%)
1	TETPY	ACN: H_2_O	24 h	88
2	CNDIPY	ACN: H_2_O	24 h	80
3	TETPY	DMSO	24 h	30
4	TETPY	DMSO: H_2_O	24 h	50
5	TETPY	ACN	24 h	45
6	TETPY	Water	24 h	35
7	TETPY	DMF	24 h	45
8	TETPY	MeOH	24 h	55
9	TETPY	EtOH	24 h	50
10^a^	—	ACN: H_2_O	24 h	NR
11^b^	TETPY	ACN: H_2_O	24 h	NR
12^c^	TETPY	ACN: H_2_O	24 h	Traces

^a^No Photocatalyst, ^b^No light, ^c^Inert atmosphere, NR No reaction.

In order to check the role of each component, we carried out hydroxylation in the absence of photocatalyst/light radiation and in both cases, no product formation was detected (Table 4, entries 10, 11). Further, we carried out the hydroxylation of 4-formylphenylboronic acid using assemblies of **TETPY** as photocatalysts under an inert atmosphere, however, the desired product was obtained in trace amount only (Table 4, entry 12). These results show that the reaction is being promoted by *in-situ* generated ROS by assemblies of **TETPY** derivative upon the natural sunlight irradiation under aerial conditions.

**Figure 17:**
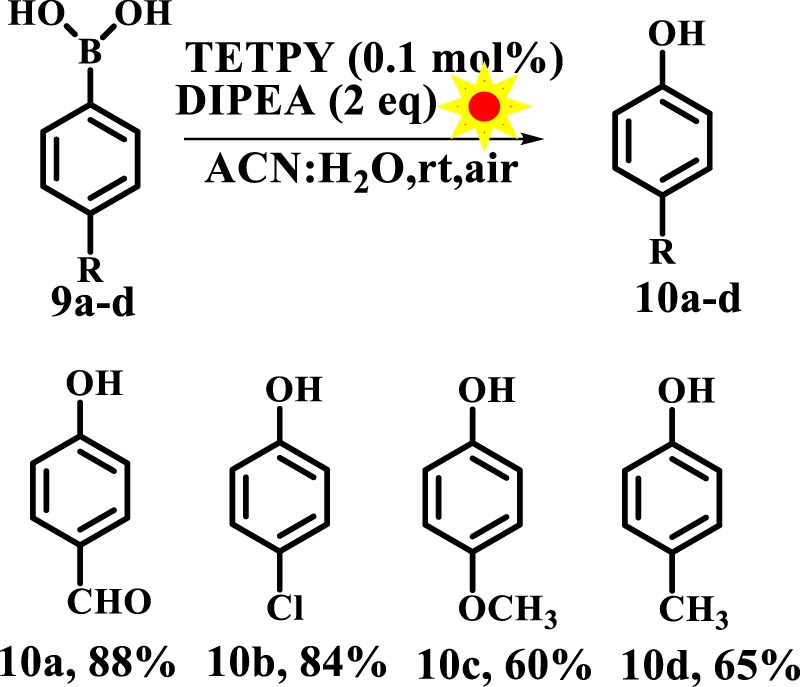
Hydroxylation of substituted boronic acid using supramolecular assemblies of **TETPY** as photocatalysts under natural sunlight and aerial conditions.

With optimized conditions in hand, the substrate scope was explored with regard to different aryl boronic acids. The aryl boronic acids with electron withdrawing groups furnished the desired product in relatively higher yields in comparison to the aryl boronic acids having electron donating groups (Fig. 17).

To confirm the role of ROS, the model reaction was carried out in the presence of singlet oxygen scavengers such as sodium azide^36^ and DABCO^37^ using **TETPY** as photocatalysts to furnish the desired products in 65% and 58% yields respectively (Fig. S30 in the Supplementary).The reaction was also carried out in the presence of superoxide scavenger such as 1,4-benzoquinone^38^ and the product was obtained in 20% yield. These studies show that superoxide radical generated through single electron transfer (SET) is the key participant in the reactions.

**Figure 18:**
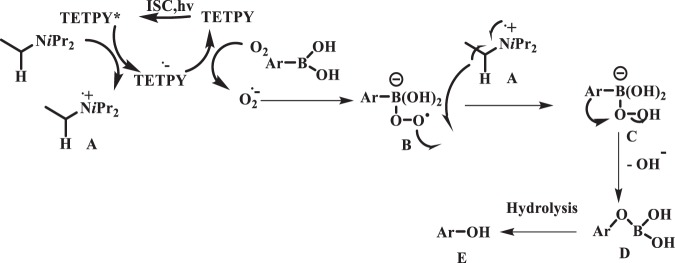
Proposed mechanism of hydroxylation of boronic acids using supramolecular assemblies of **TETPY** as photocatalysts under natural sunlight and aerial conditions.

Based upon the experimental results and literature reports, we propose the plausible mechanism for the hydroxylation of boronic acid using assemblies of **TETPY** as photocatalyst under natural sunlight irradiation as shown in Fig. 18 ^48^. The TETPY upon photoexcitation and ISC comes to triplet excited state TETPY*. Superoxide radical is being generated through the electron transfer process from DIPEA through the formation of TETPY^−^. under natural sunlight irradiation. The superoxide radical reacts with arylboronic acid to form (B). Intermediate (B) abstracted the proton from (A) gives the formation of (C). Aryl migration takes place after the removal of hydroxyl anion from intermediate (C) to generate (D). Hydrolysis of intermediate (D) gives the target product (E).

## Conclusion

We designed and synthesized supramolecular nanoassemblies of pyrazine based donor-acceptor systems **DIPY**, **TETPY**, and **CNDIPY** which form supramolecular nanoassemblies in mixed aqueous and have AIEE and ICT characteristics. The assemblies of **TETPY** and **CNDIPY** show strong absorption in the visible region with high absorption coefficients, high ROS generation abilities, low HOMO-LUMO gaps, and high photostabilities. The assemblies of **TETPY** and **CNDIPY** act as efficient photocatalysts for carrying out (a) oxidative amidation of aromatic aldehydes (b) hydroxylation of boronic acid and (c) oxidative homocoupling of benzylamines using mild conditions such as aqueous media, aerial condition and natural sunlight as a source of irradiation. All the mechanistic investigations prove the participation of *in-situ* generated ROS in the completion of the organic transformations.

## Experimental

### UV-vis and fluorescence studies

The stock solution (10^−3^ M) of CNDIPY/TETPY/DIPY derivative was prepared by dissolving 8.97 mg/15.11 mg and 8.97 mg of the compound, respectively in 10.0 mL of DMSO. 15.0 µL of this stock solution further diluted with 2985 µL of DMSO to prepare 3.0 mL solutions of derivatives (5.0 µM) and these solutions were used for each UV-vis and fluorescence experiments.

### Determination of singlet oxygen generation ability

Singlet oxygen generation ability of derivatives (DIPY, TETPY, and CNDIPY) were determined by measurement of quenching of absorbance of ABDA in the presence of photocatalysts^21^ (DIPY, TETPY, and CNDIPY) under irradiation. The solution of 5.0 µM (DIPY, TETPY and CNDIPY) in DMSO: H_2_O (1:1) mixture containing ABDA was aerated for 5 min before irradiation. The irradiation was done in a fluorescence spectrophotometer equipped with a Xe lamp at the excitation wavelength of 400 nm for 2 min. Then the absorbance of the solution was recorded with UV-vis spectrophotometer. This experiment was repeated several times and the total exposure time was 20 min. The blank experiment was performed by giving exposure of radiation to the solution of ABDA at 400 nm for 20 minutes.

### Determination of superoxide generation ability

To a solution of pyrazine derivatives (DIPY/TETPY/CNDIPY) (5.0 µM) in DMSO: H_2_O (1:1) mixture, the 50 µM solution of N,N,N′,N′-tetramethyl-p-phenylenediamine (TMPD) was added under aerobic conditions and the solution was kept under irradiation of visible light conditions 5–20 minutes (depending on pyrazine derivatives)^26^. The color and absorbance change of the solution were recorded using UV-vis spectrophotometer.

### The DPD control experiments for oxidative coupling of benzylamine and oxidative amidation of aromatic aldehydes

The DPD (N,N-diethytl-1,4-phenylenediammonium sulphate) and POD (Horseradish peroxide) were added to the reaction mixtures (oxidative coupling of benzylamines and oxidative amidation of aromatic aldehydes) in the presence of **TETPY** photocatalyst/without photocatalyst. The mixture was kept for stirring under aerial conditions in sunlight^32,38^. As the reaction proceeds, the H_2_O_2_ released *in-situ* and oxidizes DPD to DPD radical cation catalyzed by POD with intense pink coloration. The absorbance of the solution was monitored with UV-vis spectrophotometer.

## Supplementary information

Supplementary information
