# Supplementary material for: AIEE Active Nanoassemblies of Pyrazine Based Organic Photosensitizers as Efficient Metal-Free Supramolecular Photoredox Catalytic Systems
Source: Sci Rep. 2019 Jul 31;9:11142. doi: 10.1038/s41598-019-47588-5 (PMC6668430; doi:10.1038/s41598-019-47588-5)
Supplement: Supplementary file 1 — Supplementary information [file 41598_2019_47588_MOESM1_ESM.pdf]

Supplementary Information

AIEE Active Nanoassemblies of Pyrazine Based Organic Photosensitizers as Efficient Metal-Free Supramolecular Photoredox Catalytic Systems

*Shruti Dadwal, Harnimarta Deol, Manoj Kumar, Vandana Bhalla**

Department of Chemistry, UGC Sponsored Centre for Advanced Studies-II

Guru Nanak Dev University, Amritsar 143005, and Punjab, India

* *E-mail: vanmanan@yahoo.co.in*

1. General Experimental Methods and Materials

All the reagents were purchased from Aldrich and were used without further purification. HPLC grade solvents were used in UV–vis and fluorescence studies. UV–vis spectra were recorded on SHIMADZU UV-2450 spectrophotometer, with a quartz cuvette (path length 1 cm). The fluorescence spectra were recorded with HORIBA Scientific FLUOROMAX-4 spectrofluorometer. TEM study was carried out by FEI TECHNAI G^2 20 S-TWIN. The electrochemical measurements were performed at room temperature using a computer controlled potentiostat/galvanostat Autolab PGSTAT204 (Netherlands) Metrohm, equipped with FRA (frequency response analyzer) module. The NOVA software was used to collect, plot and analyze the raw data of various CV measurements. The dynamic light scattering (DLS) data were recorded with MALVERN Instruments (Nano-ZS). DFT calculations were

done by using the Gaussian 09. ^{1}H and ^{13}C NMR spectra were recorded on a Bruker Avance III HD 500 MHz and JEOL 400 MHz using $CDCl_3$ as solvent. Data are reported as follows: chemical shift in ppm (δ), multiplicity (s = singlet, d = doublet, t = triplet, m = multiplet, br = broad singlet), coupling constants J (Hz).

2. Synthesis of pyrazine derivative DIPY

To a solution of dibromopyrazine (0.4 g, 0.90 mmol) and 4-(2,2’,6’,2’’-terpyridine-4’-yl)phenyl boronic acid (0.73 g, 2.07 mmol) in anhydrous dioxane (20 mL), 2 mL aqueous solution of K_2CO_3 (0.99 g, 7.2 mmol) was added followed by the addition of [$Pd(PPh_3)_2$] (0.51 g, 0.45 mmol) as a catalyst under nitrogen atmosphere. The reaction mixture was refluxed overnight and dioxane was then removed under vacuum. The residue so obtained was treated with water and extracted with ethyl acetate three times, dried over anhydrous sodium sulphate. The solvent was removed under reduced pressure and compound was purified by column chromatography using 80:20 (ethylacetate: hexane) as an eluent to give 80% yield of the derivative **DIPY** as white solid; mp: >250°C; ^{1}H NMR (500 MHz, $CDCl_3$) δ (ppm) = 8.80 (s, 4H), 8.75 (d, *J* = 5 Hz, 4H), 8.69 (d, *J* = 5 Hz, 4H), 8.23 (d, *J* = 10 Hz, 2H), 8.03 (d, *J* = 5 Hz, 4H), 7.91-7.88 (m, 4H), 7.84-7.78 (m, 8H), 7.73 (d, *J* = 10 Hz, 4H), 7.67 (d, *J* = 5 Hz, 2H), 7.39-7.35 (m, 4H), ^{13}C NMR (CDCl3, 100 MHz, ppm) = 156.33, 156.10, 149.26, 141.40, 140.90, 137.04, 130.57, 129.34, 128.55, 127.91, 127.70, 127.20, 123.96, 121.49, 118.79. ESI-MS found to be (m/z): 897.365 correspond to parent ion peak.

3 Synthesis of pyrazine derivative TETPY

To a solution of tetrabromopyrazine (0.4 g, 0.67 mmol) and 4-(2,2’,6’,2’’-terpyridine-4’-yl)phenyl boronic acid (1.01 g, 2.88 mmol) in 20 mL anhydrous dioxane, 2 mL aqueous solution of K_2CO_3 (1.10 g, 8.04 mmol) was added followed by the addition of [$Pd(PPh_3)_2$] (0.38 g, 0.33 mmol) as a catalyst under nitrogen atmosphere. The reaction mixture was refluxed overnight and thereafter cooled to room temperature. The dioxane was removed

under vacuum to obtain solid residue. The solid was treated with water and the extracted with ethyl acetate (three time). The combined organic layers were dried over anhydrous Na_2SO_4. The solvent was removed under reduced pressure and compound was purified by column chromatography using 80:20 (ethylacetate: hexane) as an eluent to give 85% of the derivative TETPY as yellow solid; mp: >220°C; ^{1}H NMR (500 MHz, $CDCl_3$): δ (ppm) = 8.80 (s, 8H), 8.79-8.74 (m, 4H), 8.70-8.65 (m, 10 H), 8.38-8.33 (m, 2H), 8.04 (d, J = 5 Hz, 4H), 7.92-7.87 (m, 10 Hz), 7.83-7.68 (m, 14H), 7.59-7.56 (m, 2H), 7.51- 7.47 (m, 6H), 7.38-7.32 (m, 6H), ^{13}C NMR ($CDCl_3$, 100 MHz, ppm) = 156.30, 156.01, 149.25, 137.38, 136.96, 132.29, 130.59, 127.92, 127.71, 127.24, 123.98, 121.50, 118.93, 118.78. ESI-MS found to be (m/z): 1512.518 $[M+H]^+$.

4. Synthesis of pyrazine derivative CNDIPY

To a solution of dicyanopyrazine (0.4 g, 0.90 mmol) and 4-(2,2',6',2''-terpyridine-4'-yl) phenyl boronic acid (0.73 g, 2.08 mmol) in 20 mL anhydrous dioxane, 2 mL aqueous solution of K_2CO_3 (1.0 g, 7.26 mmol) was added followed by the addition of $[Pd(PPh_3)_2]$ (0.52 g, 0.45 mmol) as a catalyst under nitrogen atmosphere. The reaction mixture was refluxed overnight and thereafter cooled to room temperature. The dioxane was removed under vacuum to obtain solid residue. The solid was treated with water and the extracted with ethyl acetate (three time). The combined organic layers were dried over anhydrous Na_2SO_4. The solvent was removed under reduced pressure and compound was purified by column chromatography using 80:20 (ethylacetate: hexane) as an eluent to give 80% of the derivative CNDIPY as yellow solid; mp: >250°C; ^{1}H NMR (500 MHz, $CDCl_3$): δ (ppm) = 8.84 (s, 2H), 8.80 (s, 2H), 8.76 (t, J = 5 Hz, 4H), 8.72-8.69 (m, 4H), 8.36 (d, J = 5 Hz, 1H), 8.32 (d, J = 5 Hz, 1H), 8.16 (d, J = 5 Hz,2H), 8.04 (d, J = 10 Hz, 2H), 7.93-7.89 (m, 5H), 7.85 (d, J = 5 Hz, 1H), 7.81-7.78 (m, 3H), 7.74-7.72 (m, 4H), 7.68 (d, J = 5 Hz, 1H), 7.40-7.37 (m, 4H), ^{13}C NMR ($CDCl_3$, 125 MHz, ppm) = 156.22, 156.06, 149.24, 142.39, 137.02, 132.49, 128.09,

127.99, 127.65, 127.53, 123.99, 121.46, 118.97, 118.73. ESI-MS found to be (m/z): 936.2809 $[M+K]^+$.

5. **Preparation of photocatalyst**. (a) The 4.0 mg of TETPY dissolved in 800 μL of DMSO and 1200 μL distilled water. The 500 μL of resulting solution was used as photocatalyst for each reaction. (b) The 3.0 mg of CNDIPY/DIPY dissolved in 800 μL of DMSO and 1200 μL distilled water. The 500 μL of resulting solution was used as photocatalyst for each reaction.

6. General procedure for oxidative homocoupling of benzylamine using pyrazine derivatives as photocatalysts

The benzyl amine (**4a-d**) (0.5 mmol) in acetonitrile (ACN) (2.0 mL) in presence of CNDIPY/TETPY/DIPY (0.1 mol%) as a photocatalyst was stirred at room temperature for 6h under aerial condition in sunlight. After the completion of the reaction (monitored by TLC), the organic part was extracted with ether and the combined organic layer was dried over anhydrous sodium sulphate and distilled under reduced pressure. The products (**5a-d**) were isolated by flash chromatography. All the products were identified by ^{1}H NMR spectroscopy (Figures S40-S43).

7. General procedure for oxidative amidation of aromatic aldehydes using pyrazine derivatives as photocatalysts

The mixture of aromatic aldehyde (**6a-e**) (0.6 mmol) and pyrrolidine (**7**) (1.98 mmol) in DMSO:H_2O (1:1) mixture in presence of CNDIPY/TETPY/DIPY (0.1 mol%) as a photocatalyst was stirred at room temperature for 8h under aerial condition in sunlight. After the completion of the reaction (monitored by TLC), organic part was extracted with EtOAc. The combined organic layer was dried over anhydrous sodium sulphate and concentrate under reduced pressure. The products (**8a-e**) were purified by column chromatography using EtOAc: hexane as eluent. All the products were identified by ^{1}H NMR spectroscopy (Figures. S44-S48).

8. General procedure for oxidation of boronic acid using pyrazine derivatives as photocatalysts

The substituted boronic acids (**9a-e**) (0.5 mmol) in ACN:H_2O (2.0 mL) (1:1) solvent mixture in the presence of CNDIPY/TETPY/DIPY (0.1 mol%) as a photocatalyst was stirred at room temperature for 24h under aerial conditions in sunlight. After the completion of the reaction (monitored by TLC), organic part was extracted with EtOAc. The combined organic layer was dried over anhydrous sodium sulphate and concentrate under reduced pressure. The products (**10a-d**) were purified by column chromatography using EtOAc: hexane as eluent. All the products were identified by ^{1}H NMR spectroscopy (Figures. S49-S52).

Table S1. Comparison of the photocatalytic activity of the pyrazine derivatives for oxidative homocoupling of benzyl amines over other photocatalytic systems reported in the literature.

**Entry**	**Journals**	**Photocatalysts**	**Catalytic Loading**	**Source of Light**	**Reaction medium**	**Rxn Temp (^{0}C)**	**Time (h)**	**Oxygen Pressure**	**Yield (%)**
**1**	**Present Manuscript**	**Pyrazine based derivatives**	**0.1 mol%**	**Sunlight**	**ACN**	**RT**	**6**	**-**	**85**
2	ACS Catal. **2019**, 9, 422−430	Zn-MOF	1 mol%	660nm NIR LED	ACN	60	24	yes	62
3	ACS Catal. **2018**, 8, 6751−6759	Truxene based polymers	10 mg	sunlight	ACN	RT	4	-	90
4	*ACS Sustainable Chem. Eng*. **2017**, 5, 2562−2577	$BiVO_4$/g-C_3N4 Nanocomposite	20 mg	250 W medium pressure visible Hg Lamp	ACN	25-27	16	-	Selectivity 100
5	*Molecules* **2015**, *20*, 1941-1954	TiO_2 silicate composite	50 mg	500 W halogen lamp	ACN	40	24	1 atm	81
6	*J. Org. Chem.* **2013**, 78, 5627−5637	Iodo-Bodipy derivatives	1 mol%	Visible light	ACN	22	1	-	100
7	*Angew. Chem. Int*. Ed. **2011**, 50, 3934 –3937	TiO_2	10 mg	100 W Hg lamp	ACN	RT	9	1 atm	Conversion 99

Table S2. Comparison of the photocatalytic activity of the pyrazine derivatives for oxidative amidation of aromatic aldehydes over other photocatalytic systems reported in the literature.

Entry	Journals	Photocatalysts	Catalytic loading	Solvent	Time	Light source	Additive	O_2 pressure	Yield (%)
**1**	**Present manuscript**	**Pyrazine derivatives**	**0.1 mol%**	**DMSO: H_2O (1:1)**	**8h**	**Sunlight**	**-**	**-**	**90**
2	*Org. Lett.* **2018**, 20, 5861−5865	$Ir[df(CF_3)ppy]_2(dtbbpy)PF_6$	2 mol%	CCl_3Br, ACN	18h	LED blue light	Yes	-	75
3	*Angew. Chem*. **2018**, *57*, 4622−4626	Pd carbodicarbene complex	0.5 mol%	DMF	16h	23W CFL light	-	-	91
4	*Asian J. Org. Chem*. **2015**, 4, 533 – 536	Rose Bengal	0.01 mmol	ACN	48h	15W lamp	Yes	Air balloon	56
5	*Adv. Synth. Catal*. DOI: 10.1002/adsc.201600138	Ag/g-C3N4 nanocomposite	5 mol%	THF	30h	25W CFL	-	-	92
6	*Org. Lett.* **2014**, 16, 5812−5815	Phenazine ethosulfate	1-2 mol%	THF	16h	24W household lamp	-	-	81

Table S3. Comparison of the photocatalytic activity of the pyrazine derivatives for hydroxylation of boronic acid over other photocatalytic systems reported in the literature

Entry	Journals	Photocatalysts	Catalytic Loading	Source of light	Rxn medium	O_2 Pressure	Additive	Time (h)	yield (%)
**1**	**Present manuscript**	**Pyrazine derivatives**	**0.1 mol%**	**Sunlight**	**ACN:H_2O**	**-**	**DIPEA**	**24**	**88**
2	*Org. Lett*. **2018**, *20*, 4979−4983	Rose Bengal	5 mol%	12W blue LED	EtOH	-	Et_3N	12	95
3	*ACS Catal*. **2018**, 8, 5313−5322	Cationic carbazolic networks	2 mol%	23 W White LED	DMF	-	DIPEA	48	76
4	*Adv. Synth.* Catal. **2018**, 360, 1–8	C_{70} fullerene	0.05 mol%	Blue LED (6W)	$CHCl_3$	O_2 balloon	DIPEA	12	99
5	*J. Org. Chem*. **2017**, *82*, 5236−5241	N-substituted 3(10H)-acridones	1 mol%	6 W blue LED	H_2O	O_2 balloon	DIPEA	17	95
6	*ACS Catal*. **2015**, 5, 2250−2254	Carbazolic organic framework	2 mol%	14 W household lamp	DMF	-	Et_3N	24	94
7	*J. Am. Chem. Soc*. **2013**, 135, 13286−13289	Methylene Blue	1 mol%	Visible light	MeCN:H_2O	-	DIPEA	7	94
8	*Angew. Chem.* **2012**, *124*, 808 –812	$[Ru(bpy)_3Cl_2].6H_2O$	2 mol%	36 W fluorescence lamp	DMF	-	DIPEA	48	90

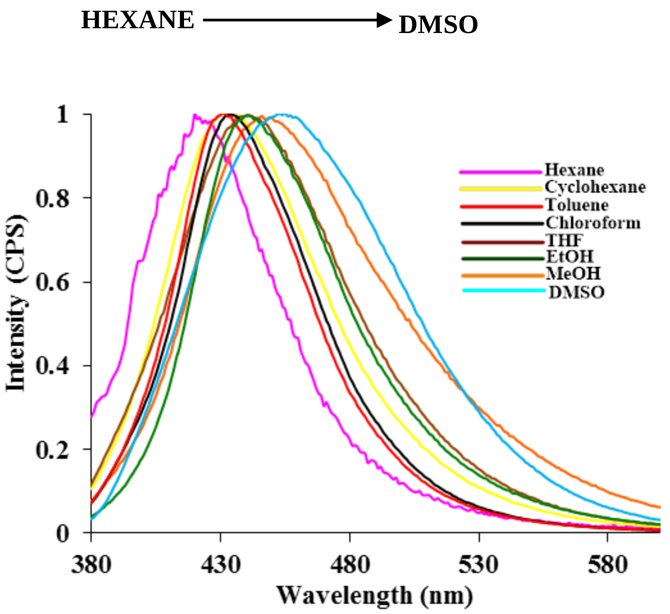

Figure S1. Normalized fluorescence spectra of **DIPY** (5μM) in different solvents from non-polar (Hexane) to polar solvent (DMSO).

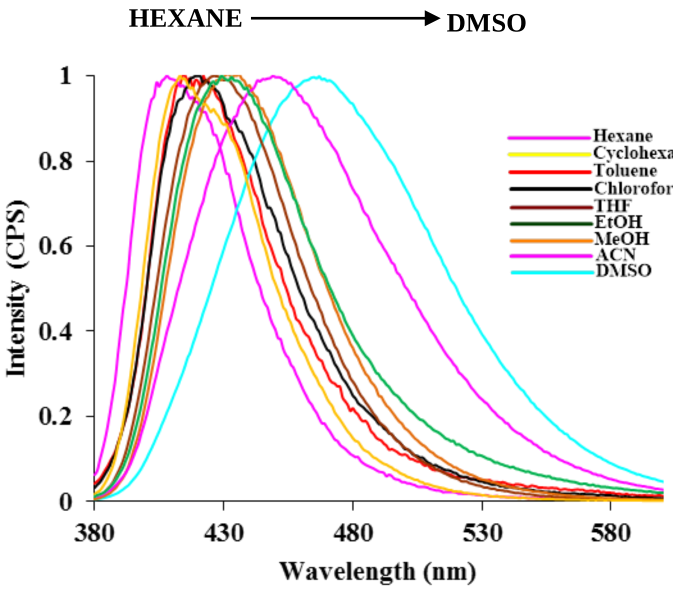

Figure S2. Normalized fluorescence spectra of **TETPY** (5μM) in different solvents from non-polar (Hexane) to polar solvent (DMSO).

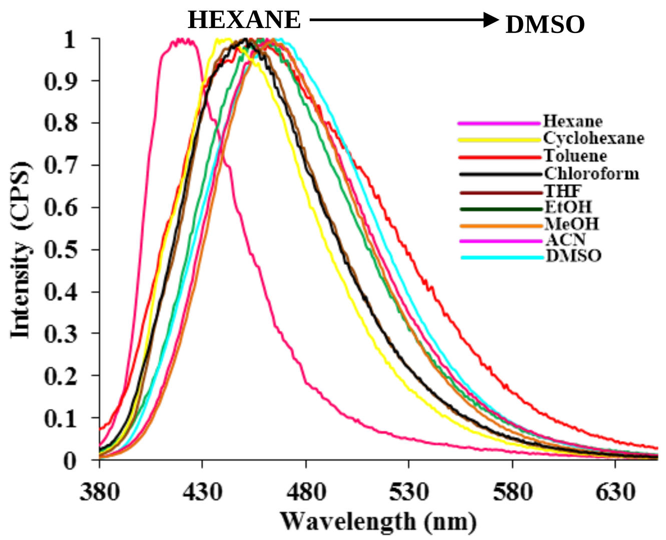

Figure S3. Normalized fluorescence spectra of **CNDIPY** (5μM) in different solvents from non-polar (Hexane) to polar solvent (DMSO).

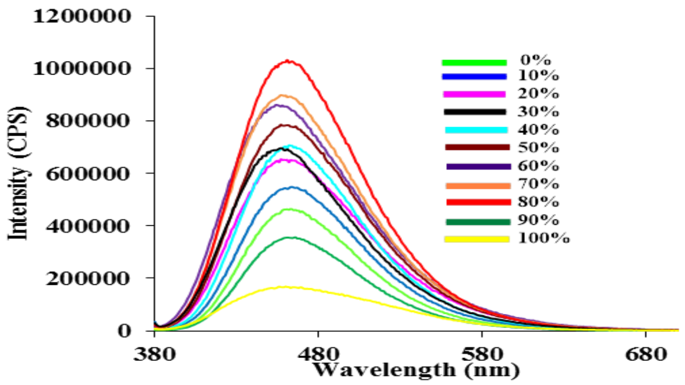

Figure S4. Fluorescence spectra of **TETPY** (5μM) in different H_2O:DMSO fractions, λ_{ex} = 360 nm.

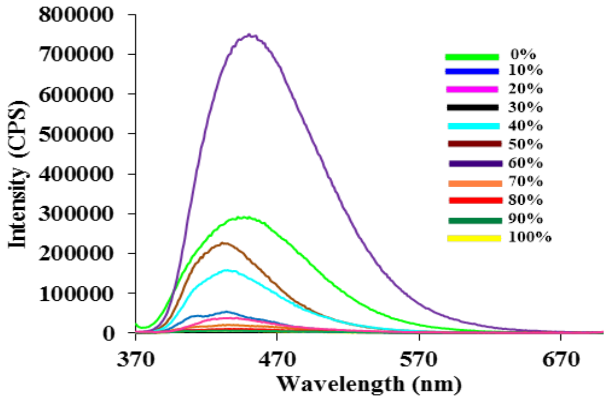

Figure S5. Fluorescence spectra of **DIPY** (5μM) in different H_2O:DMSO fractions λ_{ex} = 360 nm.

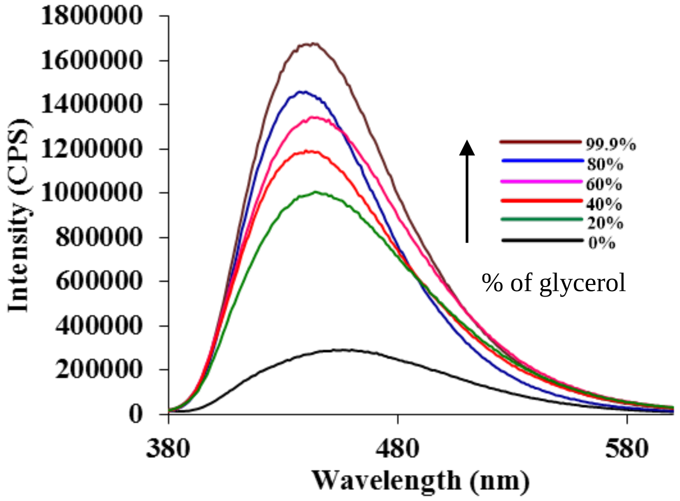

Figure S6. Fluorescence spectra of **DIPY** (5μM) in different fractions of glycerol and DMSO, λ_{ex} = 360 nm

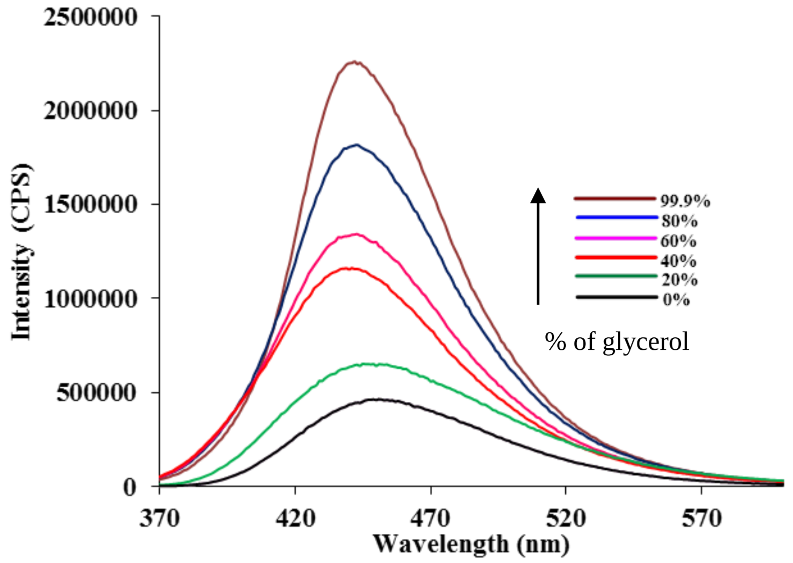

Figure S7. Fluorescence spectra of **TETPY** (5μM) in different fractions of glycerol and DMSO, λ_{ex} = 360 nm.

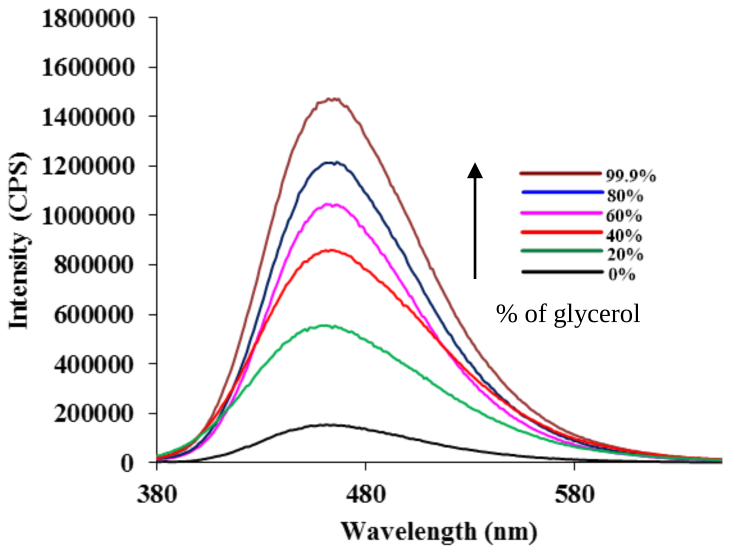

Figure S8. Fluorescence spectra of **CNDIPY** (5μM) in different fractions of glycerol and DMSO, λ_{ex} = 370 nm.

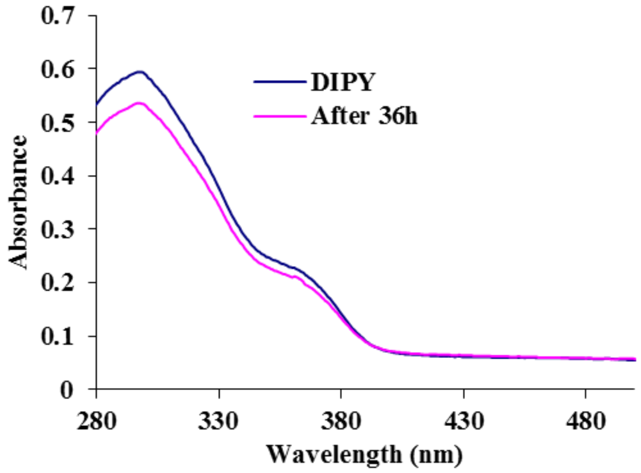

Figure S9. UV-vis spectra of derivative **DIPY** before and after irradiation of 36h.

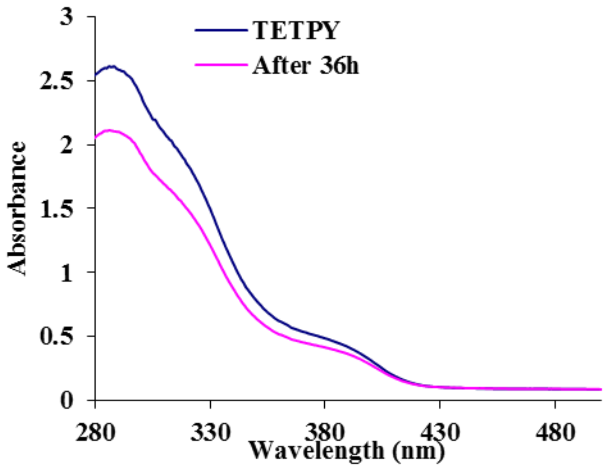

Figure S10. UV-vis of derivative **TETPY** before and after irradiation of 36h.

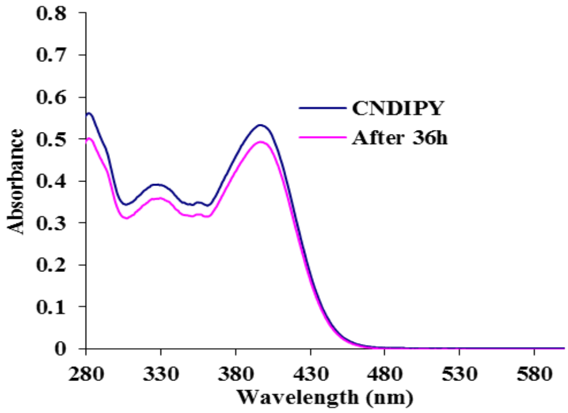

Figure S11. UV-vis spectra of derivative **CNDIPY** before and after irradiation of 36h.

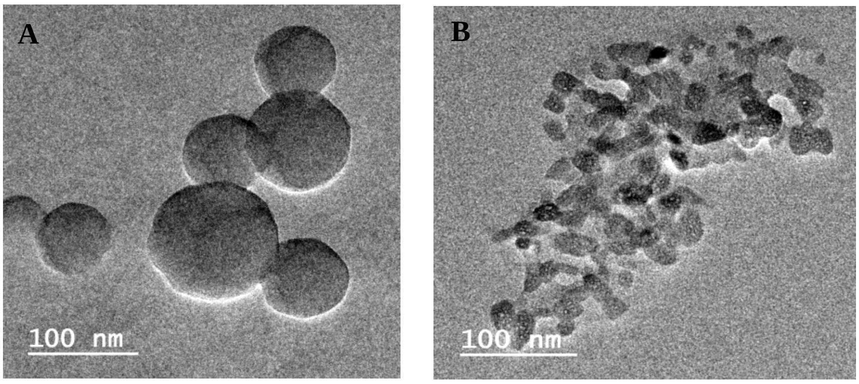

C

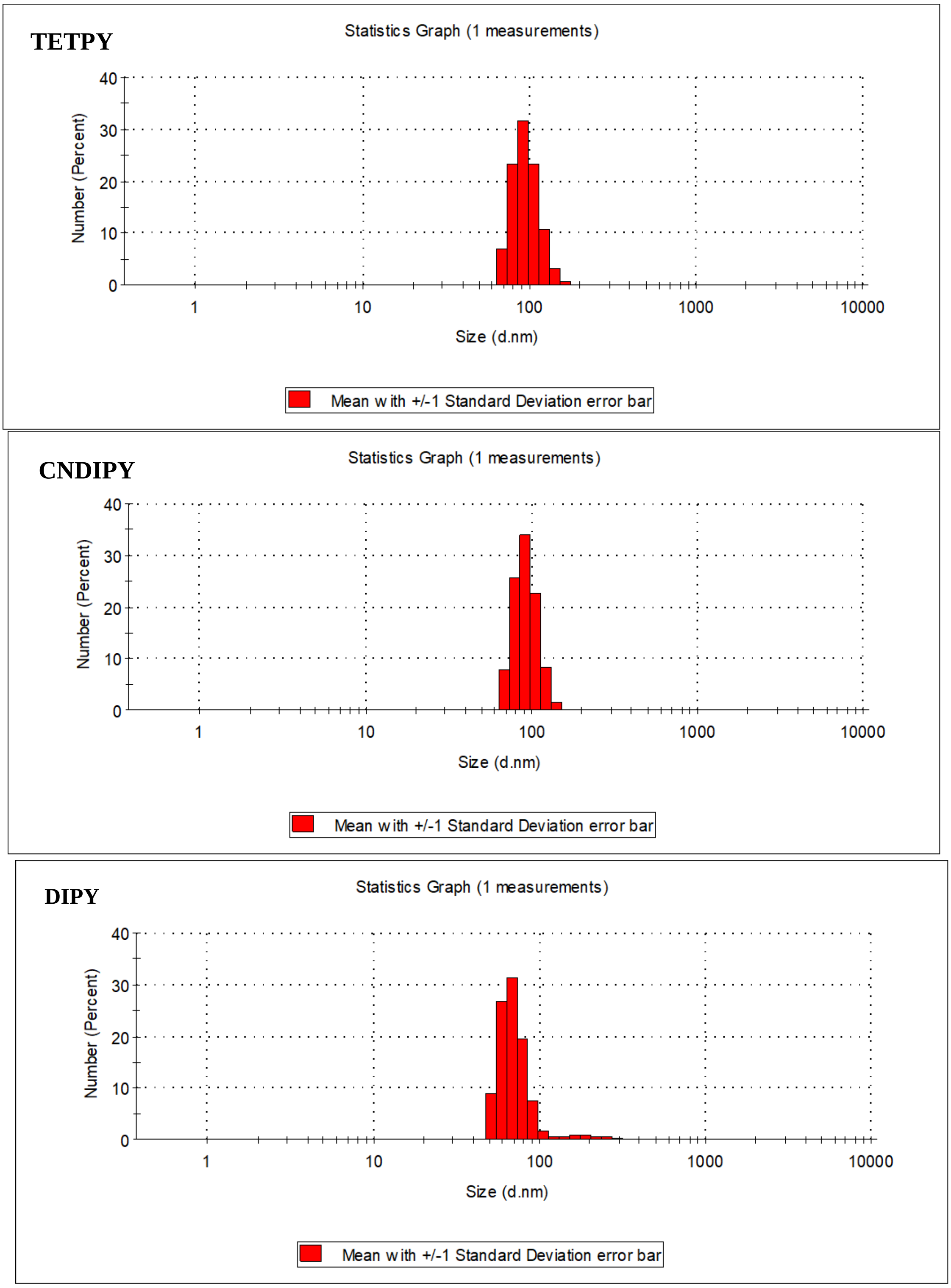

Figure S12. (A) TEM image showed the spherical nanoassemblies of **TETPY**. (B) TEM image showed the irregular nanoassemblies of **CNDIPY**. (C) DLS spectra of nanoassemblies of **TETPY**, **CNDIPY** and **DIPY** showing the particle size 50-100 nm

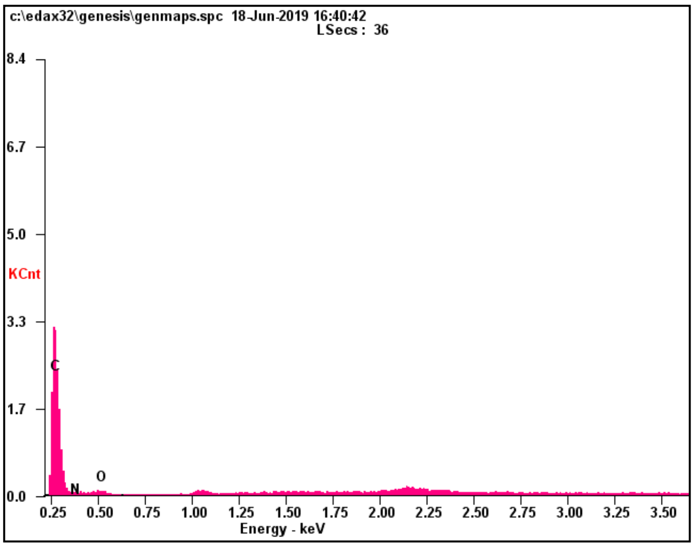

Element	***Wt%***	***At%***
CK	79.32	82.33
NK	14.06	12.51
OK	06.62	05.16
Matrix	Correction	ZAF

Figure S13. EDX spectra of TETPY derivative

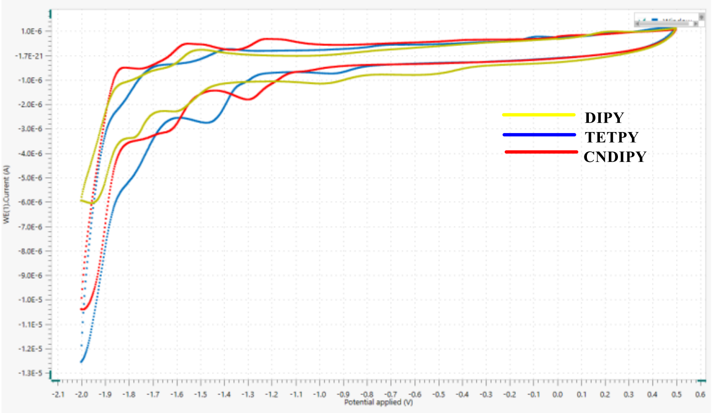

Figure S14. Cyclic voltammogram of pyrazine derivatives under N_2 saturated ACN. The potential was scanned at 100 mV s^{-1} using platinum (working), Ag/AgCl (reference) and platinum wire (counter) electrodes with (0.1 M) tetrabutylammonium hexafluorophosphate ($TBAPF_6$) as supporting electrolyte in ACN.

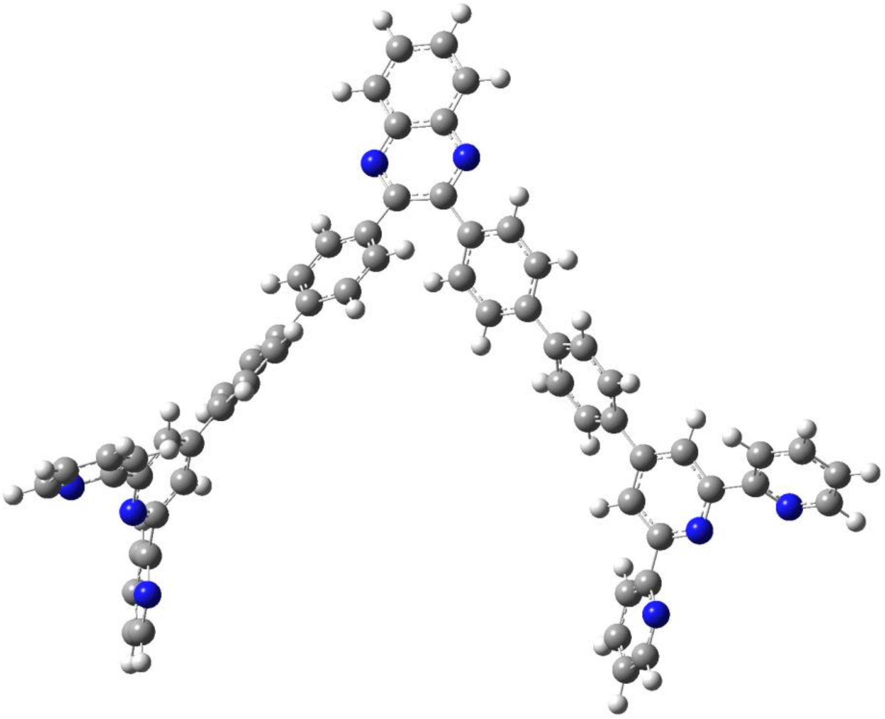

Figure S15. Optimised structure of **DIPY** by using TD-DFT (Gaussian 09/B3LYP/ 6-31G(d,p)) basic set.

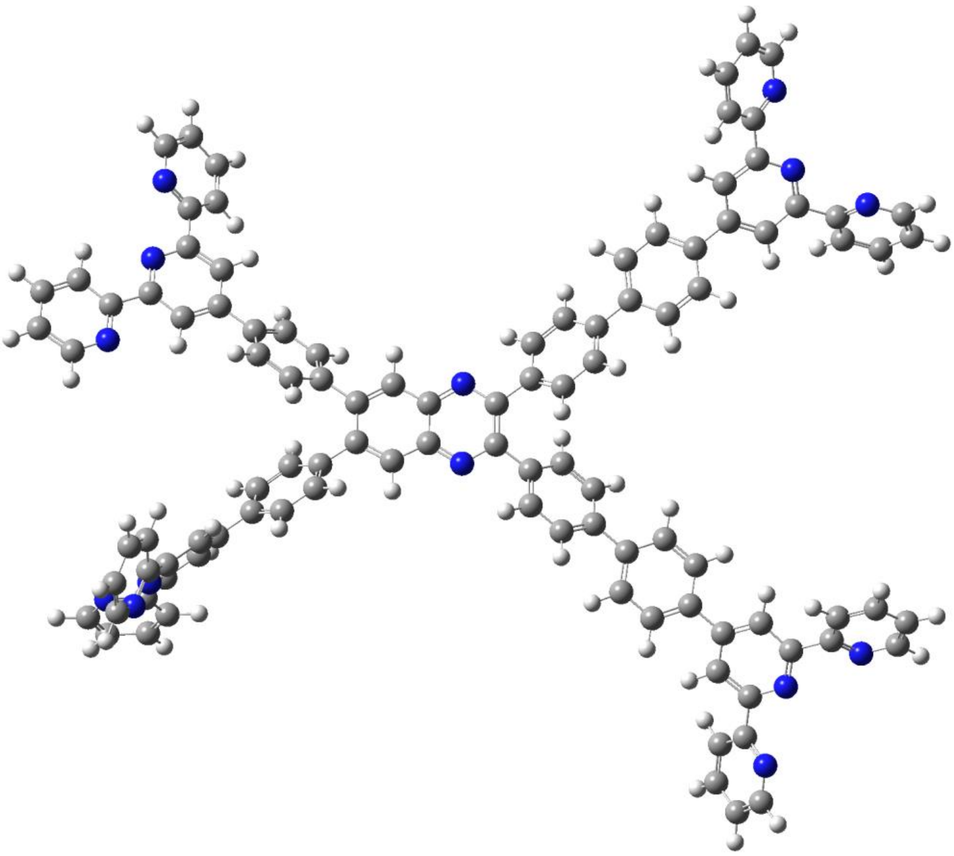

Figure S16. Optimised structure of **TETPY** by using TD-DFT (Gaussian 09/B3LYP/ 6-31G(d,p)) basic set.

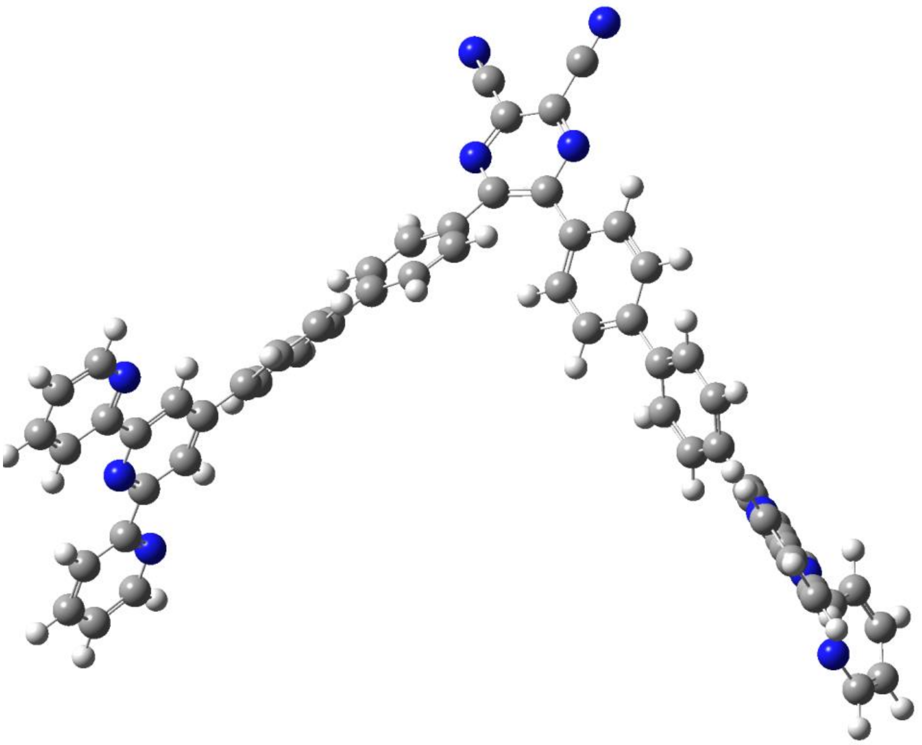

Figure S17. Optimised structure of **CNDIPY** by using TD-DFT (Gaussian 09/B3LYP/ 6-31G(d,p)) basic set.

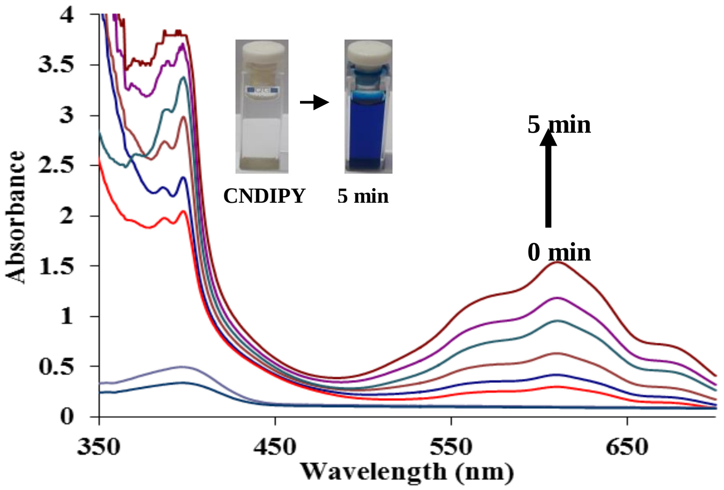

Figure S18. Absorption spectral changes of derivative **CNDIPY** (0.02 mM) in the presence of MV^{2+} (0.2 mM) and TEOA (50 mM) in DMSO:H_2O under room light and inert atmosphere.

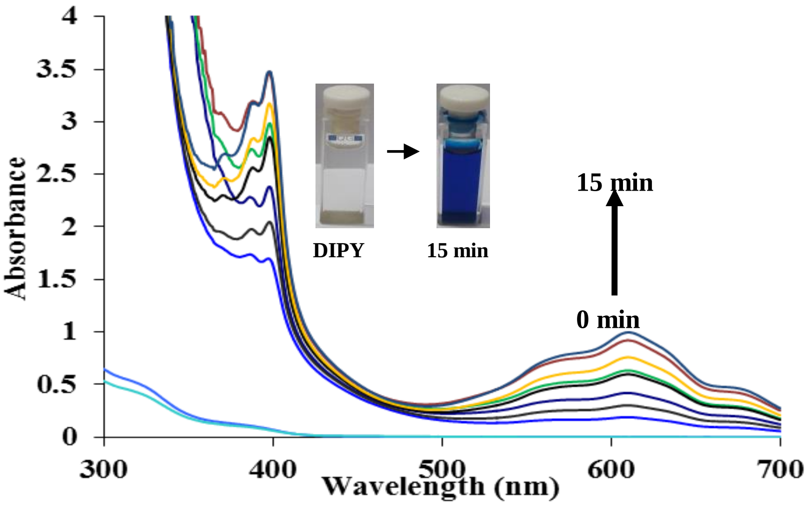

Figure S19. Absorption spectral changes of derivative **DIPY** (0.02 mM) in the presence of MV^{2+} (0.2 mM) and TEOA (50 mM) in DMSO:H_2O under room light and inert atmosphere.

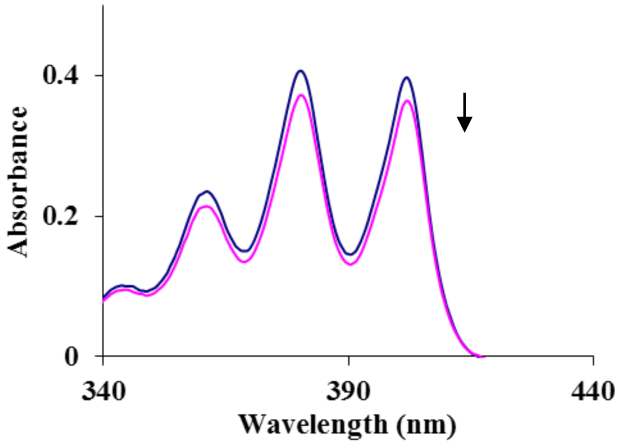

Figure S20. UV-vis spectra of ABDA before and after under irradiation of visible light for 20 minutes.

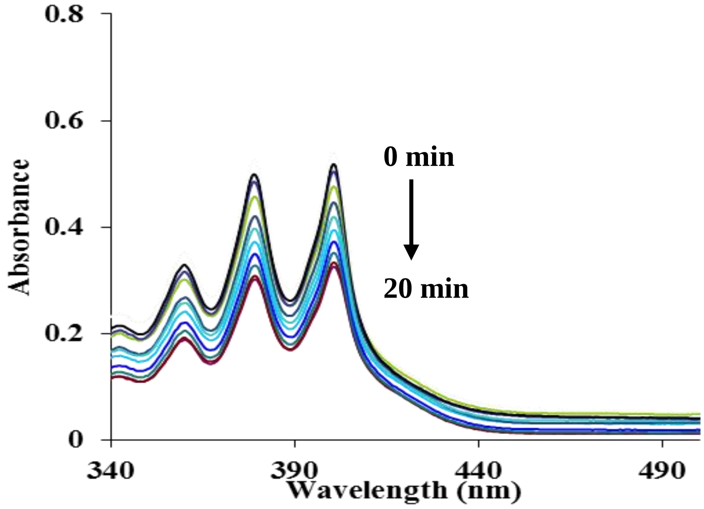

Figure S21. UV-vis spectra of ABDA in presence of monomeric form of **CNDIPY** under irradiation of visible light.

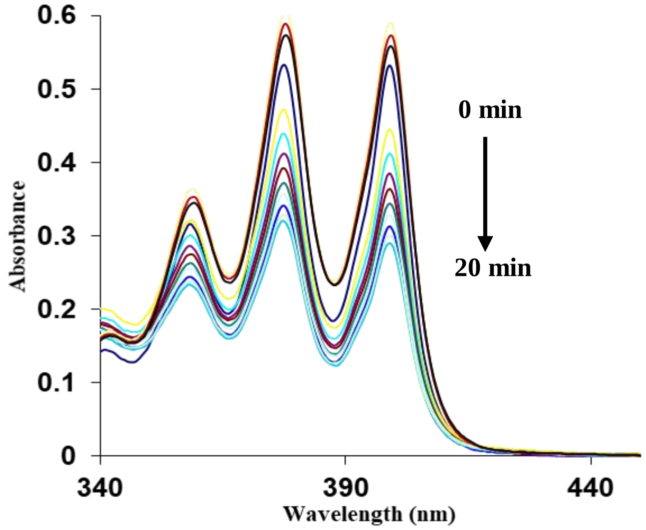

Figure S22. UV-vis spectra of ABDA in presence of nanoassemblies of **TETPY** under irradiation of visible light.

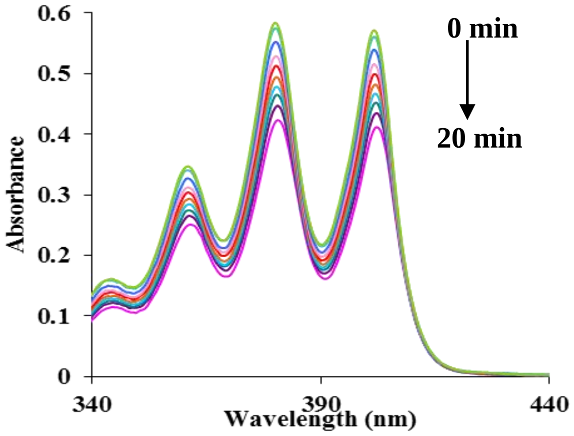

Figure S23. UV-vis spectra of ABDA in presence of monomeric form of **TETPY** under irradiation of visible light.

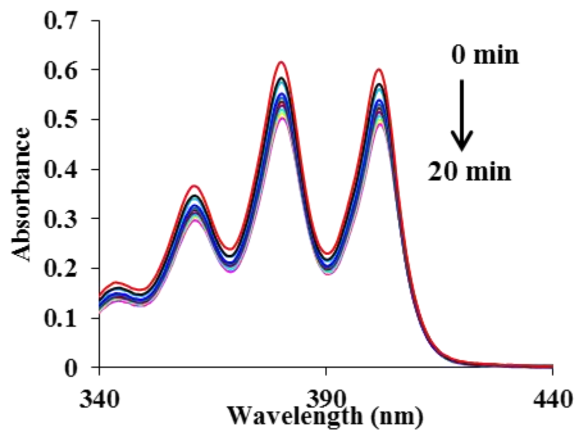

Figure S24. UV-vis spectra of ABDA in presence of nanoassemblies of **DIPY** under irradiation of visible light.

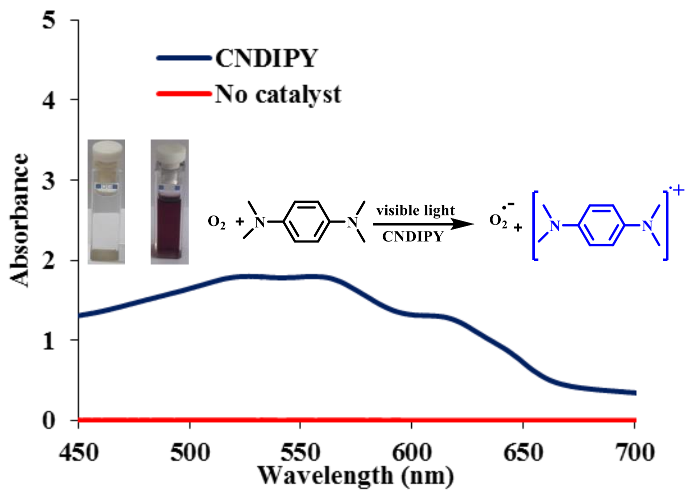

Figure S25. UV−vis absorption spectra and photographs of the cationic radical species of N,N,N′,N′-tetramethyl-p-phenylenediamine generated by assemblies of **CNDIPY** in the presence of visible light and oxygen.

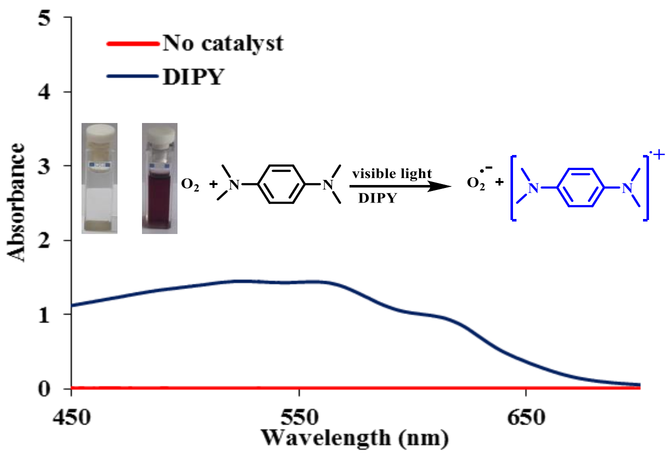

Figure S26. UV−vis absorption spectra and photographs of the cationic radical species of N,N,N′,N′-tetramethyl-p-phenylenediamine generated by assemblies of **DIPY** in the presence of visible light and oxygen.

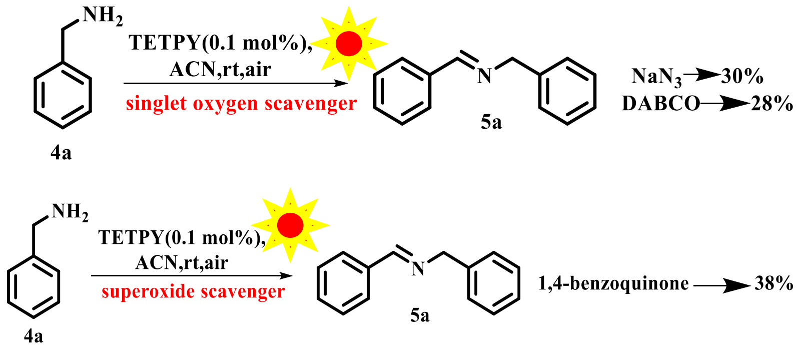

Figure S27. Oxidative coupling of benzylamine in the presence of ROS quenchers using **TETPY** as photocatalysts under natural sunlight and aerial conditions.

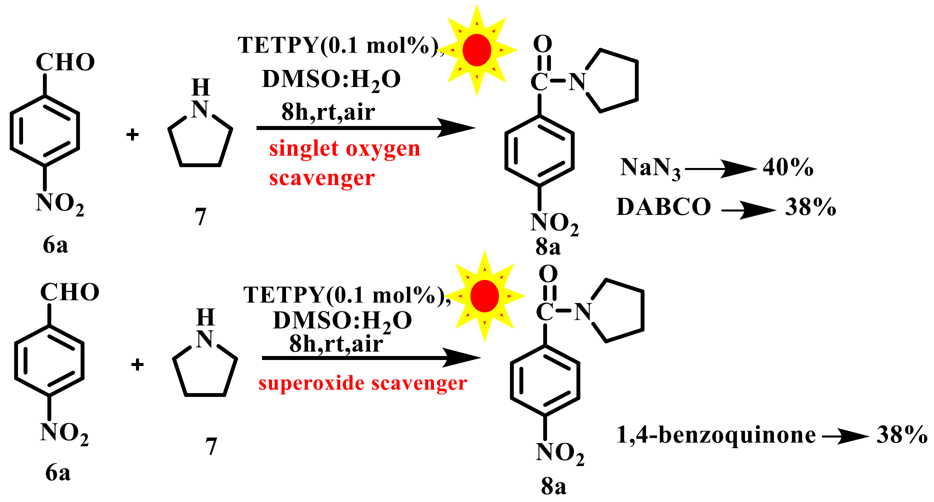

Figure S28. Oxidative amidation of 4-nitrobenzaldehyde in the presence of ROS quenchers with nanoassemblies of **TETPY** under natural sunlight and aerial conditions.

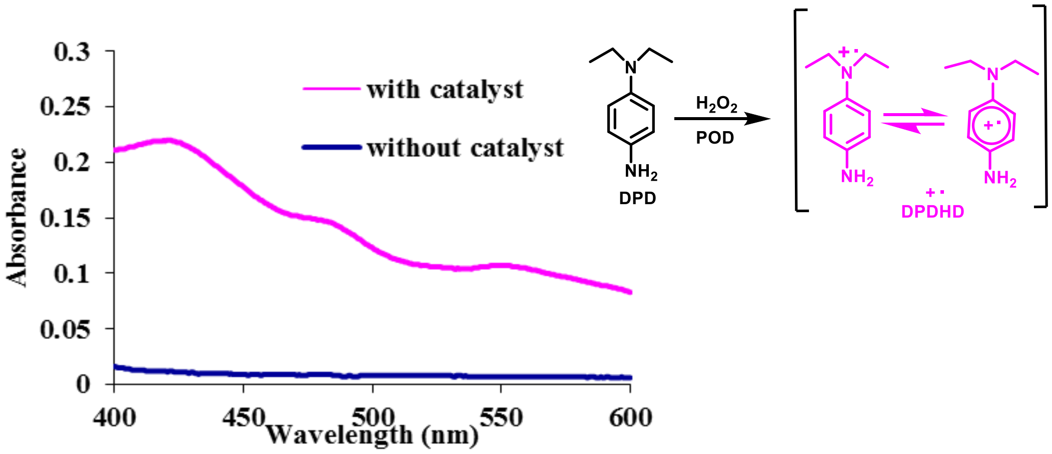

Figure S29. UV- vis absorption spectra of oxidative amidation reaction system with or without catalyst after the addition of DPD and POD.

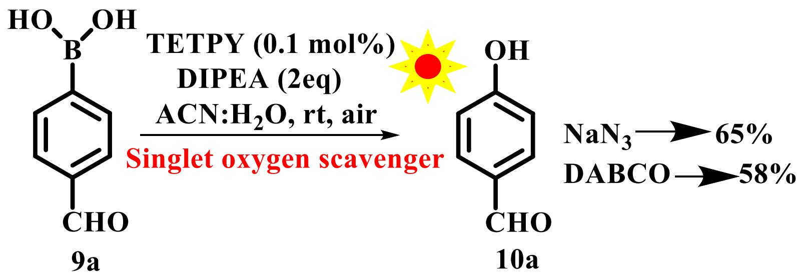

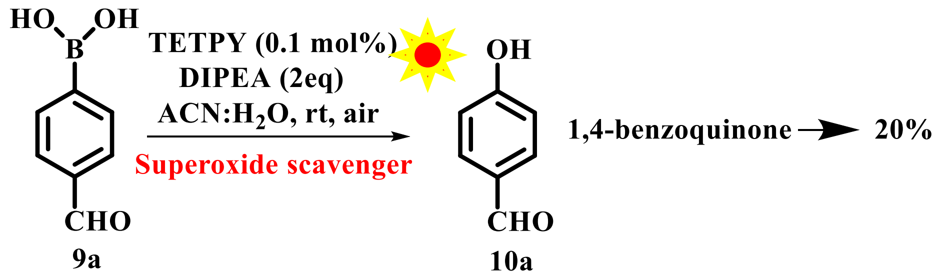

Figure S30. Hydroxylation of 4-formylphenylboronic acid in the presence of ROS quenchers using nanoassemblies of **TETPY** under natural sunlight irradiation and aerial conditions.

Compound 5a[1].^{1}H NMR (500 MHz, $CDCl_3$): δ (ppm) = 8.33 (s, 1H), 7.76 (d, *J* = 5 Hz, 2H), 7.37-7.35 (m, 3H), 7.32-7.29 (m, 4H), 7.24-7.21 (m, 1H), 4.78 (s, 2H).

Compound 5b[1]. ^{1}H NMR (500 MHz, $CDCl_3$): δ (ppm) = 8.24 (s, 1H), 7.69 (d, *J* = 10 Hz, 2H), 7.22 (d, *J* = 10 Hz, 2H), 6.89-6.84 (m, 4H), 4.68 (s, 2H), 3.76 (s, 3H), 3.73 (s, 3H).

Compound 5c[1]. ^{1}H NMR (500 MHz, $CDCl_3$): δ (ppm) = 8.34 (s, 1H), 7.71 (d, *J* = 10 Hz, 2H), 7.39 (d, J = 10 Hz, 2H), 7.31 (d, *J* = 10 Hz, 2H), 7.27-7.25 (m, 2H), 4.76 (s, 2H).

Compound 5d[1]. ^{1}H NMR (500 MHz, $CDCl_3$): δ (ppm) = 8.34 (s, 1H), 7.66 (d, *J* = 10 Hz, 2H), 7.22-7.21 (m, 4H), 7.15 (d, 2H, J = 5 Hz), 4.77 (s, 2H), 2.38 (s, 3H), 2.33 (s, 3H).

Compound 8a[2]. ^{1}H NMR (500 MHz, $CDCl_3$): δ (ppm) = 8.26 (d, *J* = 10 Hz, 2H,), 7.67 (d, *J* = 10Hz, 2H), 3.66 (t, *J* = 5 Hz, 2H), 3.37 (t, *J* = 5 Hz, 2H), 2.01-1.96 (m, 2H), 1.94-1.90 (m, 2H).

Compound 8b[2]. ^{1}H NMR (500 MHz, $CDCl_3$): δ (ppm) = 7.54 (d, *J* = 5 Hz, 2H), 7.40 (d, *J* = 10 Hz, 2H), 3.63 (t, *J* = 5 Hz, 2H), 3.41 (t, *J* = 5 Hz, 2H), 1.97-1.94 (m, 2H), 1.90-1.85 (m 2H).

Compound 8c[2]. ^{1}H NMR (500 MHz, $CDCl_3$): δ (ppm) = 7.46 (d, *J* = 10 Hz, 2H), 7.36 (d, *J* = 10 Hz, 2H), 3.62 (t, *J* = 7.5 Hz, 2H), 3.40 (t, *J* = 5 Hz, 2H), 1.98-1.94 (m, 2H), 1.90-1.86 (m, 2H).

Compound 8d[2]. ^{1}H NMR (500 MHz, $CDCl_3$): δ (ppm) = 7.71 (d, *J* = 5 Hz, 2H), 7.61 (d, *J* = 10 Hz, 2H), 3.65 (t, *J* = 7.5Hz, 2H), 3.37 (t, *J* = 5 Hz, 2H), 2.00-1.96 (m, 2H), 1.94-1.89 (m, 2H).

Compound 8e[2]. ^{1}H NMR (500 MHz, $CDCl_3$): δ (ppm) = 7.52 (d, *J* = 5 Hz, 2H), 6.90 (d, *J* = 5 Hz, 2H), 3.83 (s, 3H), 3.64 (t, *J* = 7.5 Hz, 2H), 3.48 (t, *J* = 7.5 Hz, 2H), 1.95-1.94 (m, 2H), 1.88-1.87 (m, 2H).

Compound 10a[3]. ^{1}H NMR (500 MHz, $CDCl_3$): δ (ppm) = 9.86 (s, 1H), 7.81 (d, *J* = 5 Hz, 2H), 6.97 (d, *J* = 10 Hz, 2H), 6.58 (s, 1H).

Compound 10b[3]. ^{1}H NMR (500 MHz, $CDCl_3$): δ (ppm) = 7.19 (d, *J* = 10 Hz, 2H), 6.77 (d, *J* = 10 Hz, 2H), 5.65 (br, s, 1H).

Compound 10c[3]. ^{1}H NMR (500 MHz, $CDCl_3$): δ (ppm) = 6.80-6.76 (m, 4H), 5.12 (br, s, 1H), 3.77 (s, 3H).

Compound 10d[3]. ^{1}H NMR (500 MHz, $CDCl_3$): δ (ppm) = 7.06 (d, *J* = 10 Hz, 2H), 6.78 (d, *J* = 10 Hz, 2H), 5.74 (s, 1H), 2.31 (s, 3H).

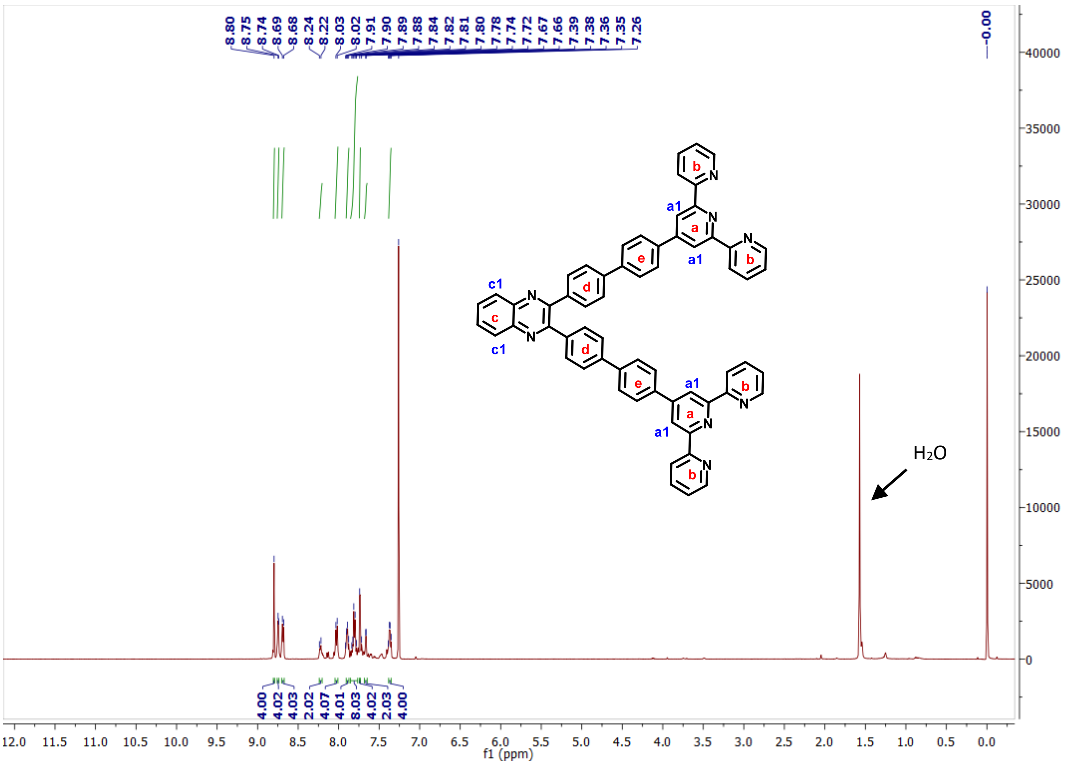

Figure S31. ^{1}H NMR Spectra ($CDCl_3$, 500 MHz, ppm) of compound **DIPY.**

S. No.	NMR Peaks of DIPY	Corresponds to
1	Peaks at 8.80 ppm	Characteristic singlet of a1 protons of ring a.
2	8.23 ppm	Characteristic doublet of c1 protons of ring c.
3	7.39-7.35 and 7.73 ppm	Ring d.
4	8.75, 8.69, 8.03, 7.91-7.88, 7.84-7.88, 7.64 and 7.84-7.88 ppm	Ring b, e and c.

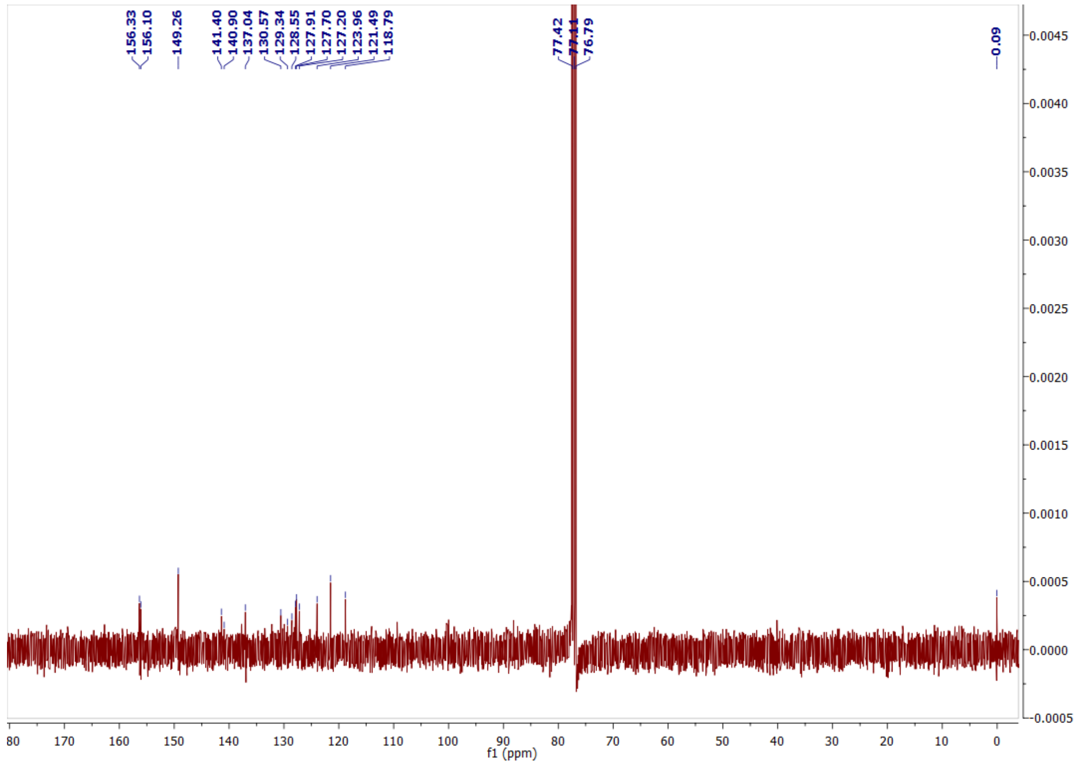

Figure S32. ^{13}C NMR Spectra ($CDCl_3$, 100 MHz, ppm) of compound **DIPY.**

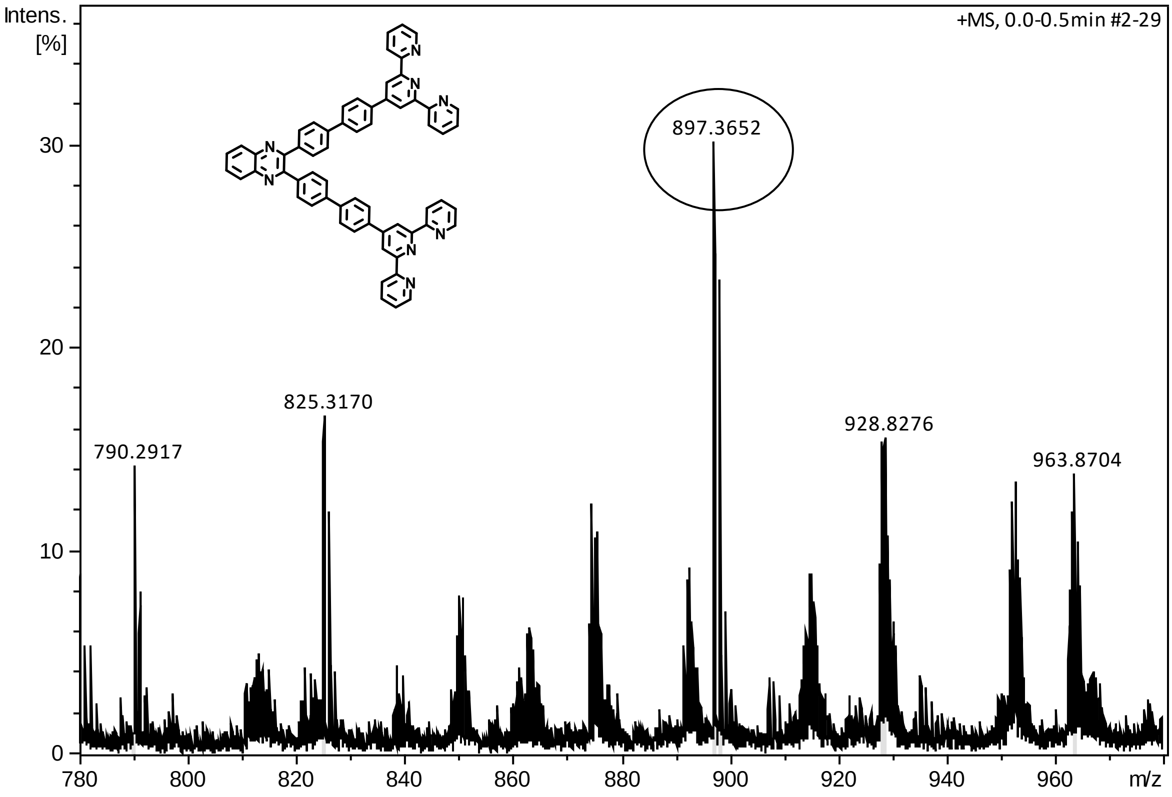

Figure S33. ESI-MS spectrum of **DIPY.**

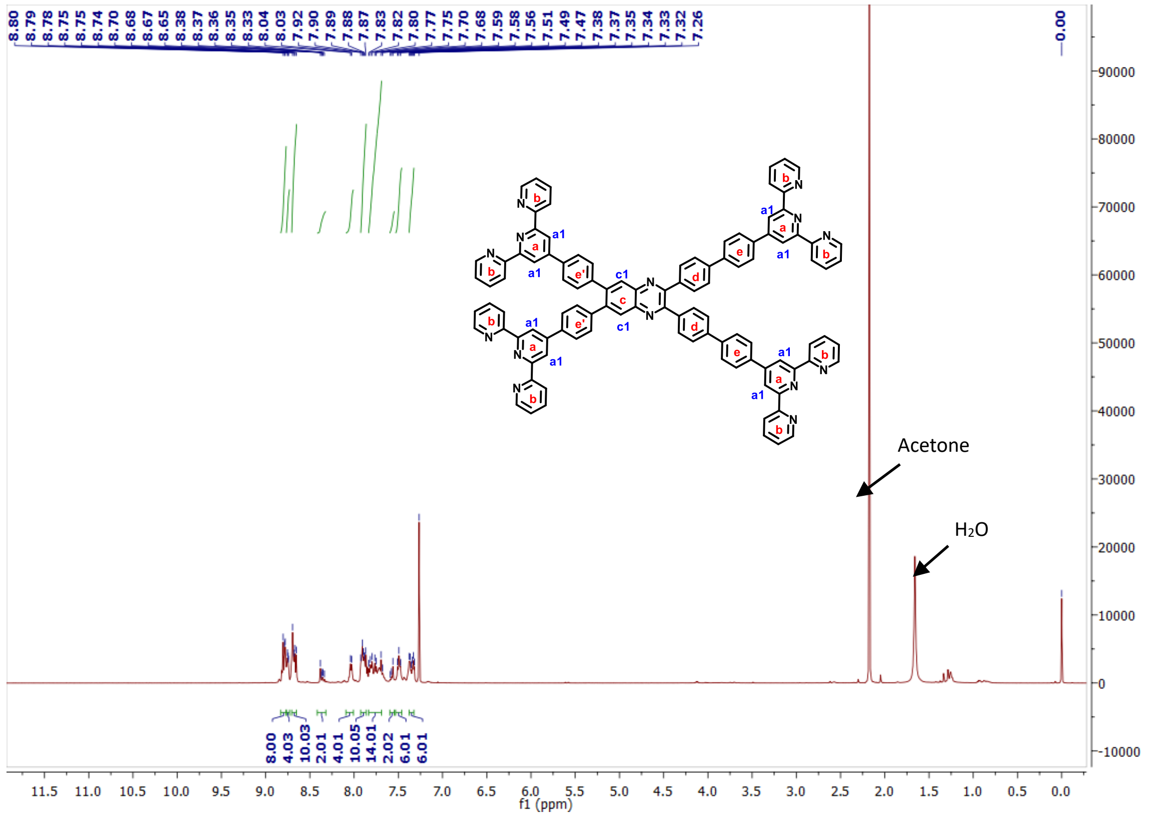

Figure S34. ^{1}H NMR Spectra ($CDCl_3$, 500 MHz, ppm) of compound **TETPY.**

S. No.	NMR Peaks of TETPY	Corresponds to
1	8.80 ppm	Characteristic singlet of a1 protons of ring a.
2	8.38-8.33 ppm	Characteristics doublet of c1 protons of ring c.
3	8.79-8.74, 8.70-8.65, 8.04, 7.92-7.87, 7.83-7.68, 7.59-7.56, 7.51-7.47 and 7.38-7.32 ppm	Ring b, c, d, e and e'.

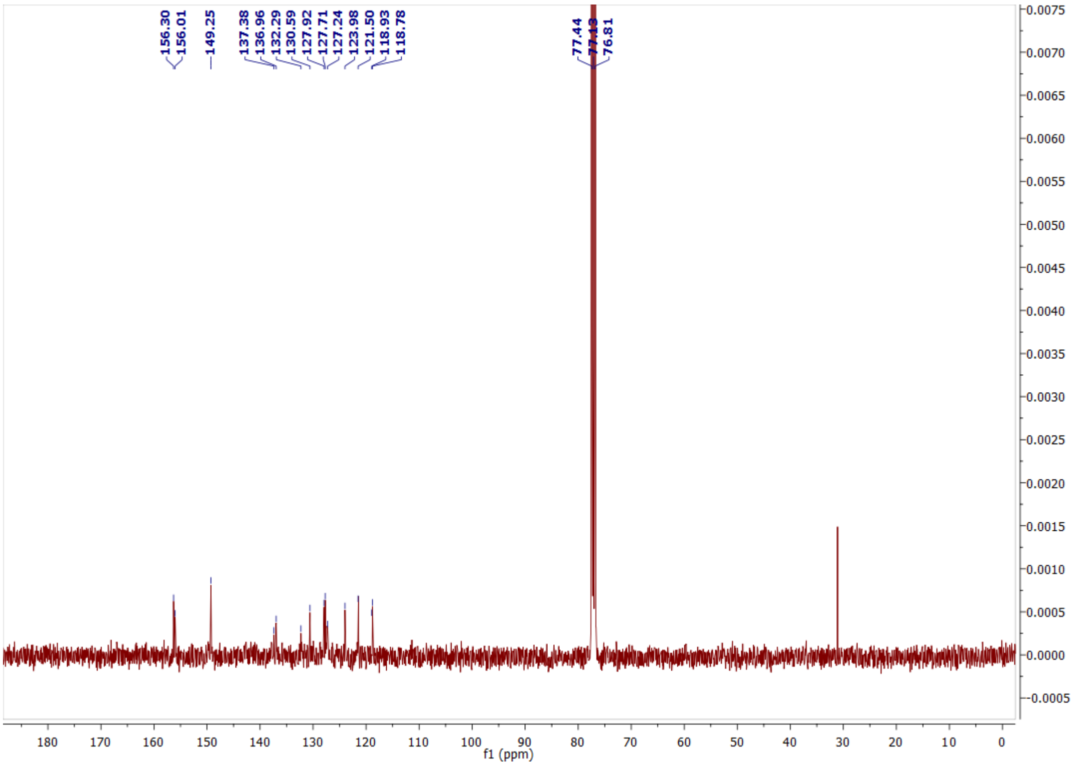

Figure S35. ^{13}C NMR Spectra ($CDCl_3$, 100 MHz, ppm) of compound **TETPY.**

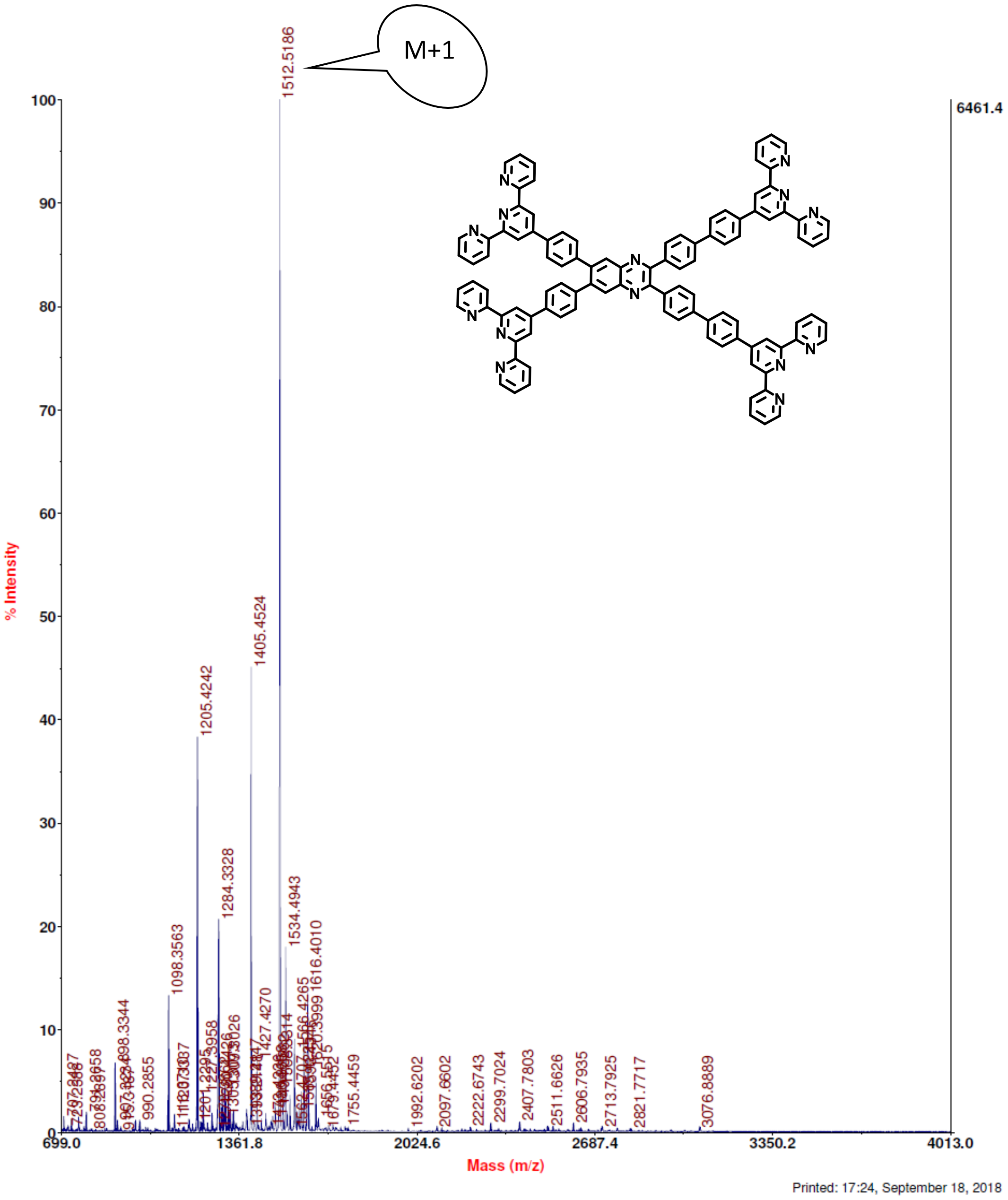

Figure S36. ESI-MS spectrum of **TETPY.**

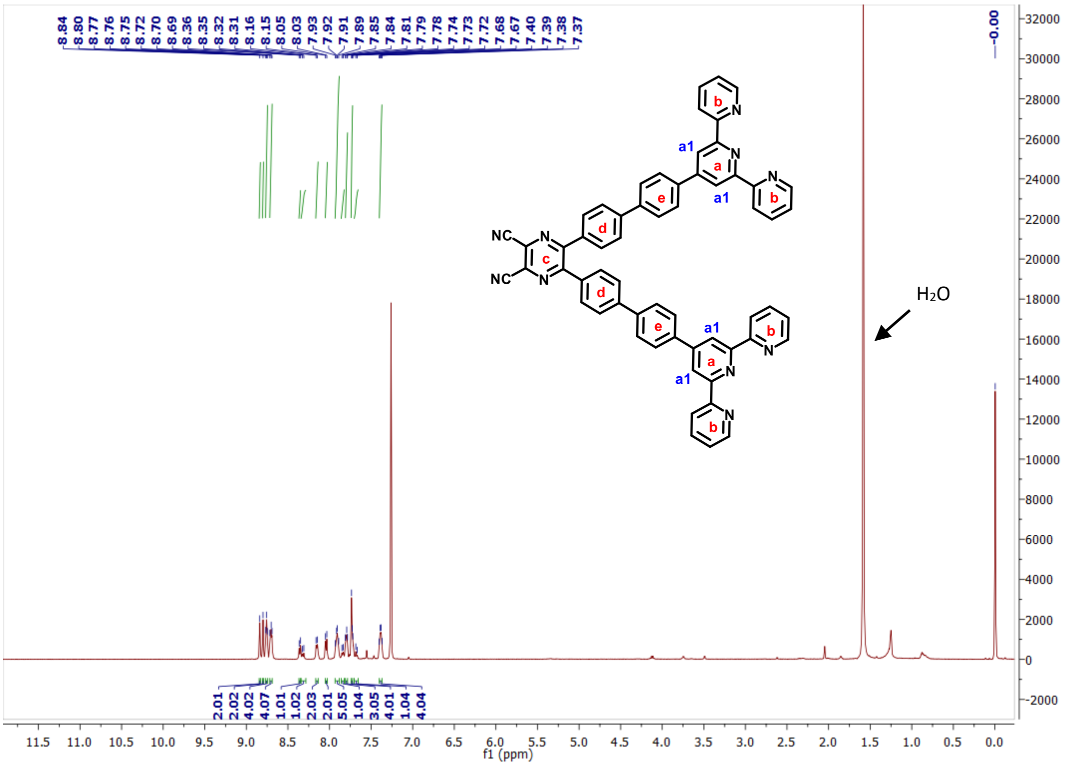

Figure S37. ^{1}H NMR Spectra ($CDCl_3$, 500 MHz, ppm) of compound **CNDIPY.**

S. No.	NMR Peaks of CNDIPY	Corresponds to
1.	8.84-8.80 ppm	Characteristic two singlets of a1 protons of ring a.
2.	7.74-7.72 and 7.40-7.37 ppm	Ring d.
3.	8.76, 8.72-8.69, 8.36, 8.32, 8.16, 8.04, 7.93-7.89,7.85 and 7.74-7.72 ppm	Ring b and e.

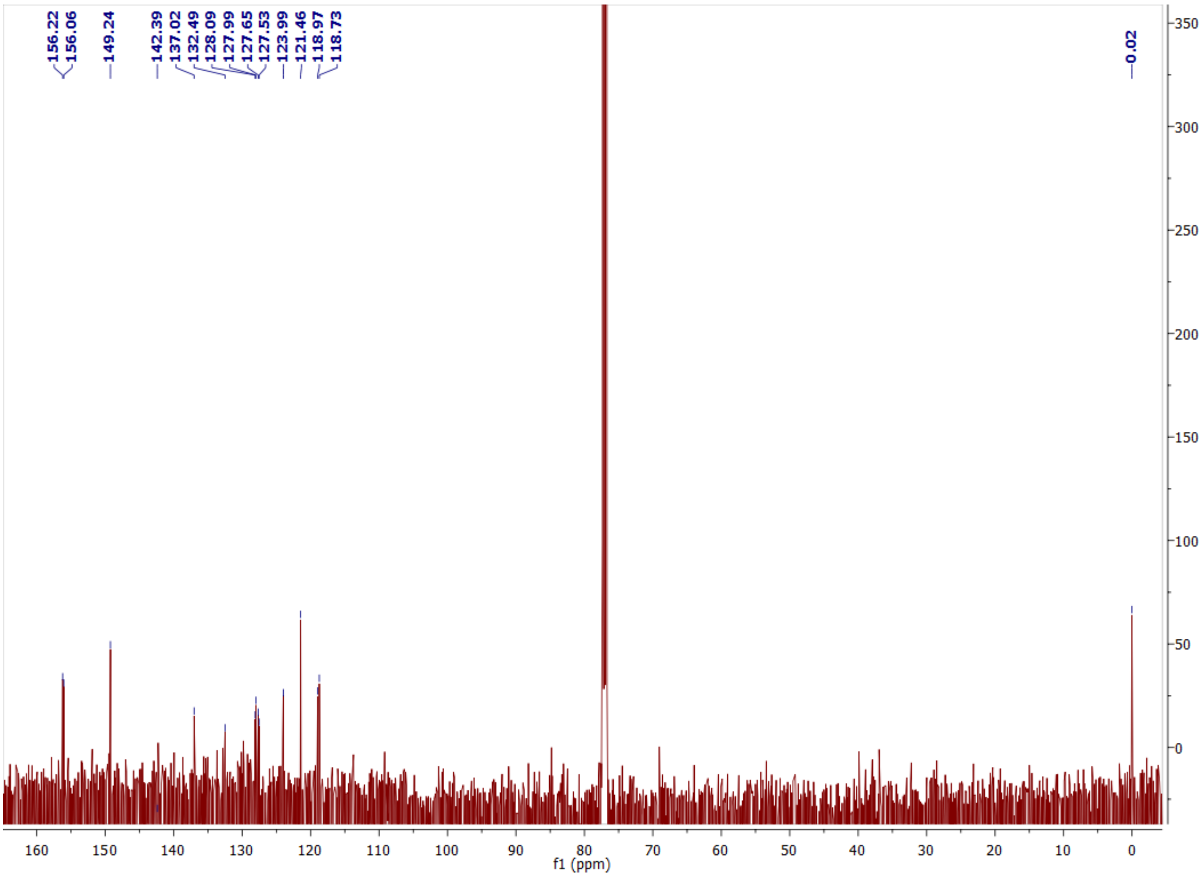

Figure S38. ^{13}C NMR Spectra ($CDCl_3$, 125 MHz, ppm) of compound **CNDIPY**

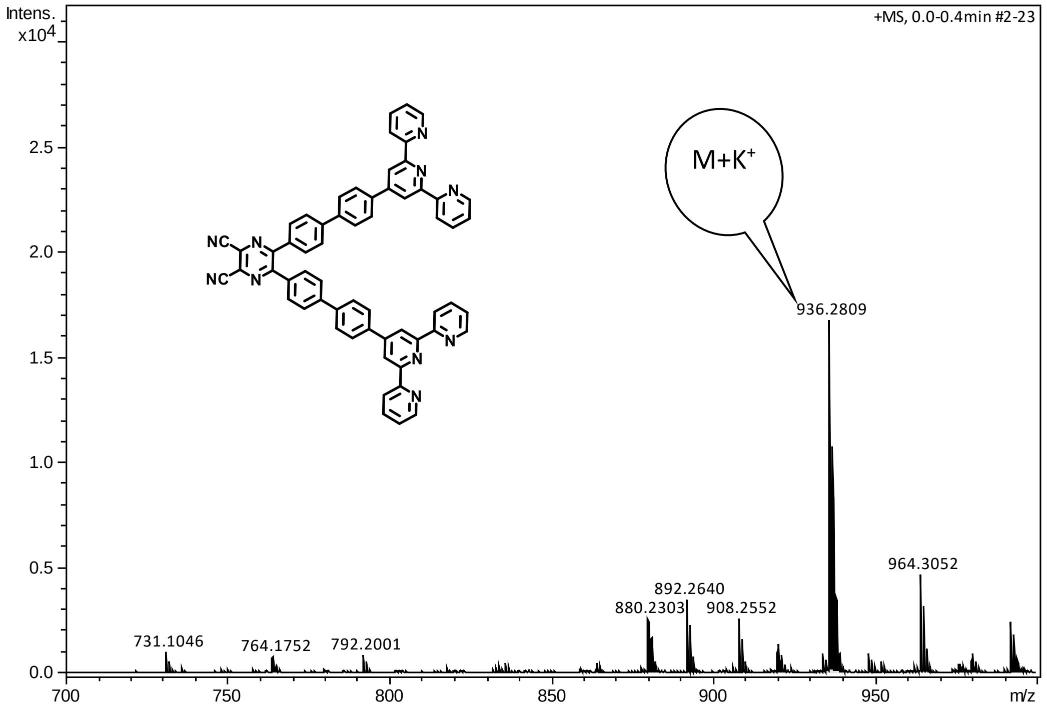

Figure S39. ESI-MS spectrum of **CNDIPY.**

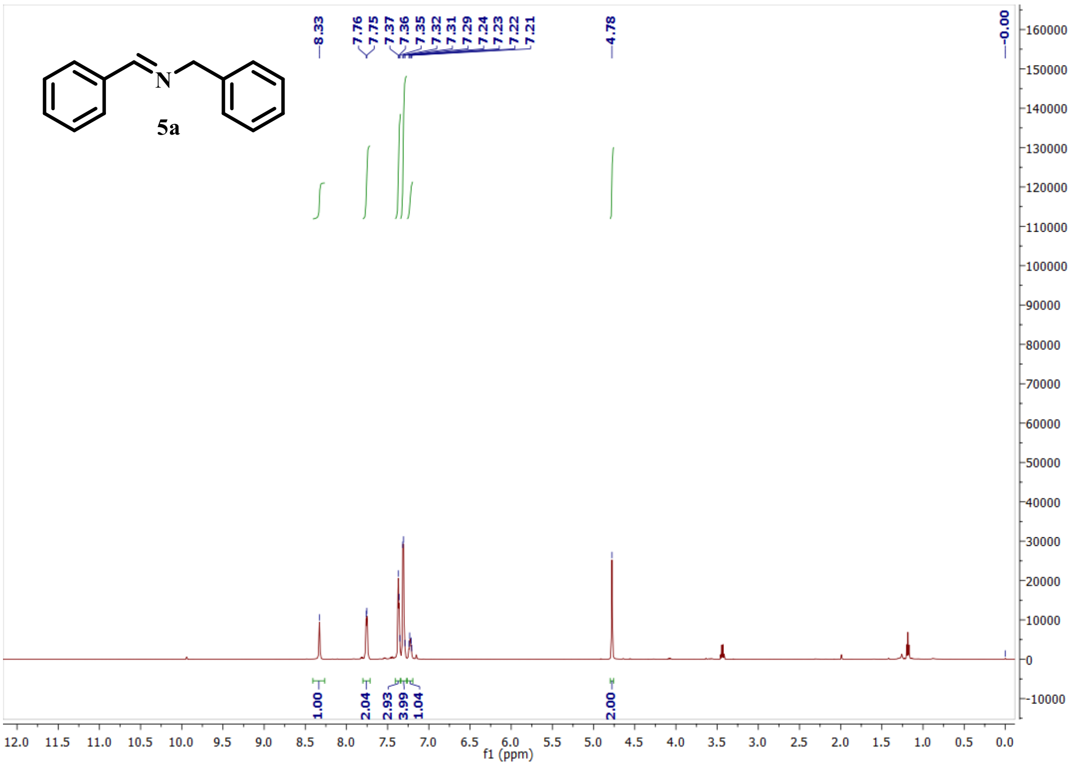

Figure S40. ^{1}H NMR Spectra ($CDCl_3$, 500 MHz, ppm) of compound **5a.**

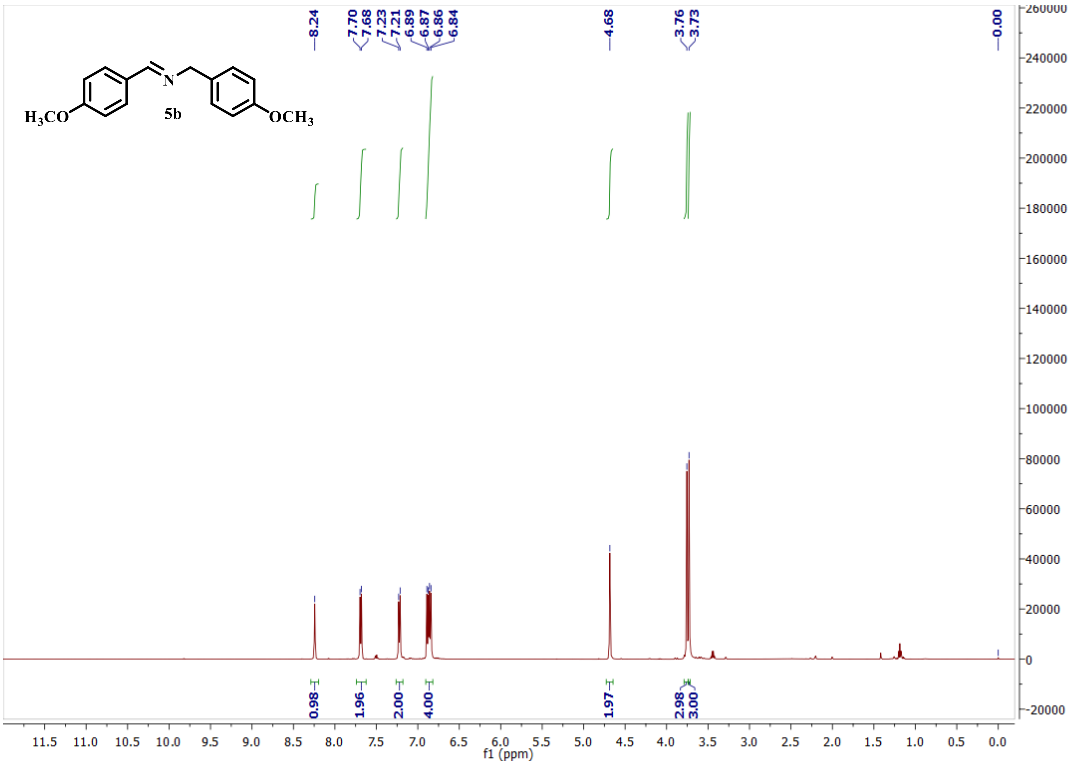

Figure S41. ^{1}H NMR Spectra ($CDCl_3$, 500 MHz, ppm) of compound **5b.**

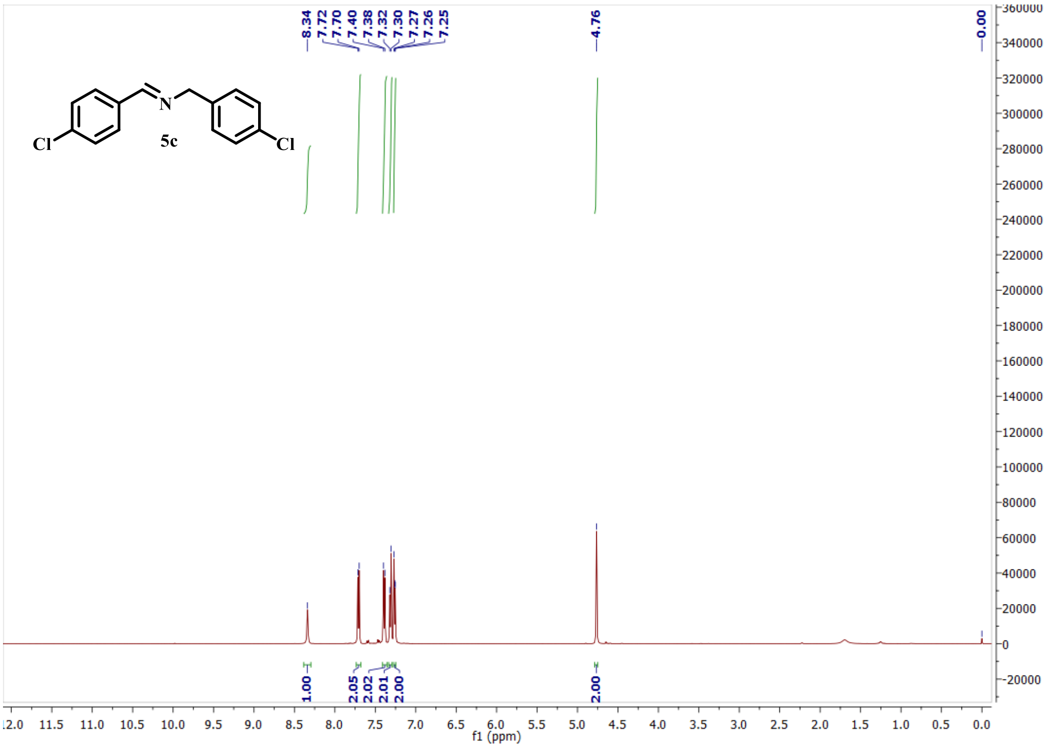

Figure S42. ^{1}H NMR Spectra ($CDCl_3$, 500 MHz, ppm) of compound **5c.**

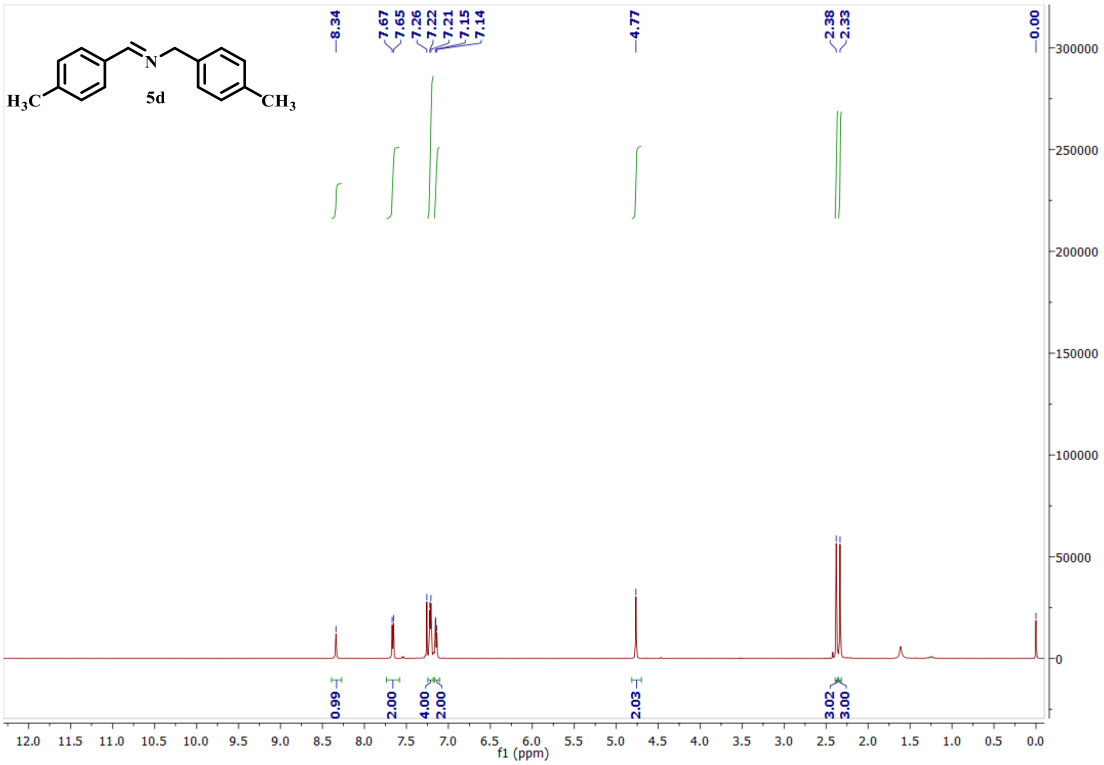

Figure S43. ^{1}H NMR Spectra ($CDCl_3$, 500 MHz, ppm) of compound **5d.**

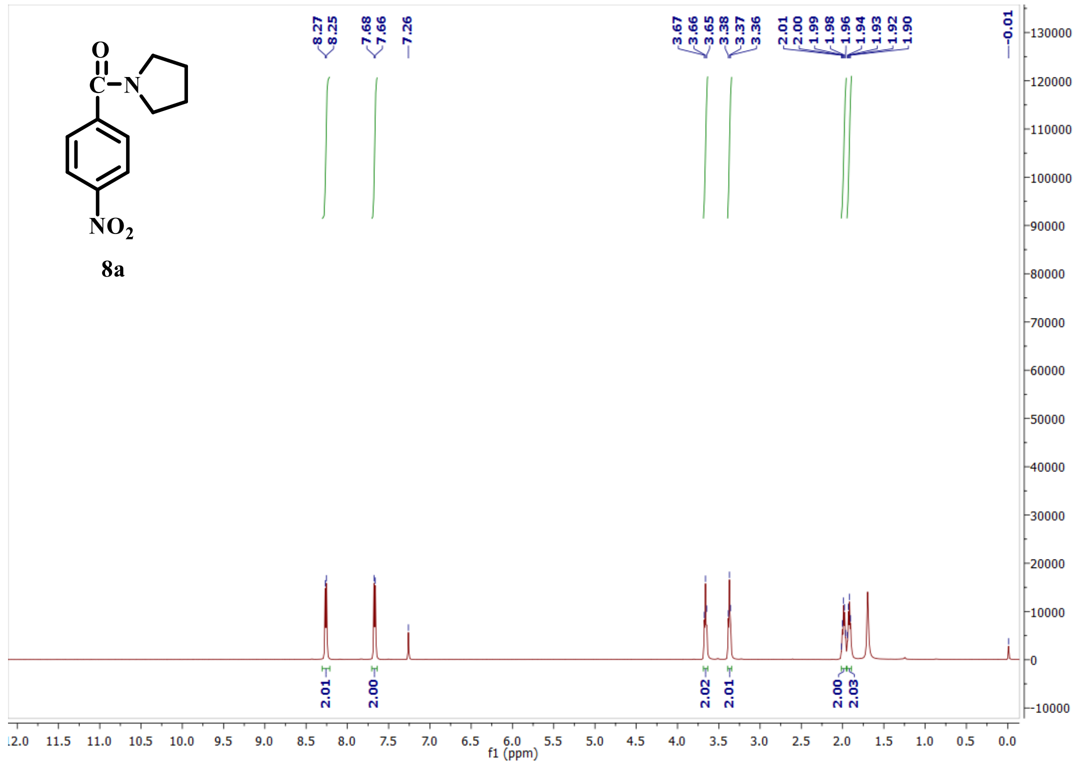

Figure S44. ^{1}H NMR Spectra ($CDCl_3$, 500 MHz, ppm) of compound **8a.**

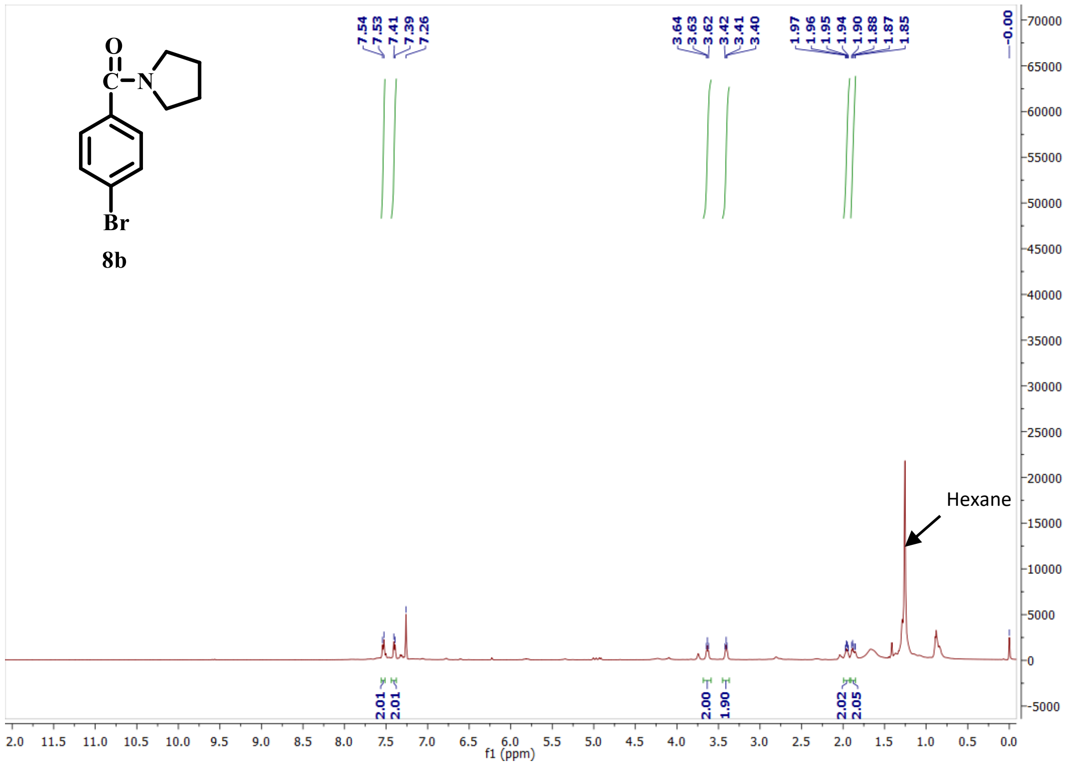

Figure S45. ^{1}H NMR Spectra ($CDCl_3$, 500 MHz, ppm) of compound **8b.**

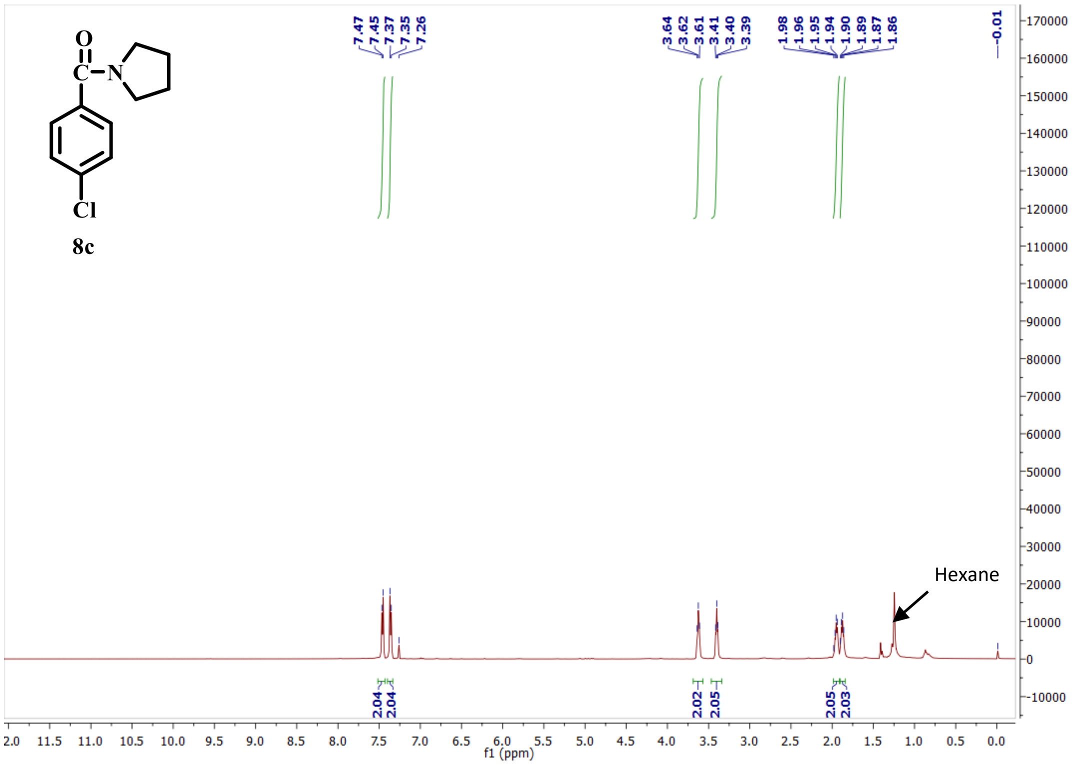

Figure S46. ^{1}H NMR Spectra ($CDCl_3$, 500 MHz, ppm) of compound **8c.**

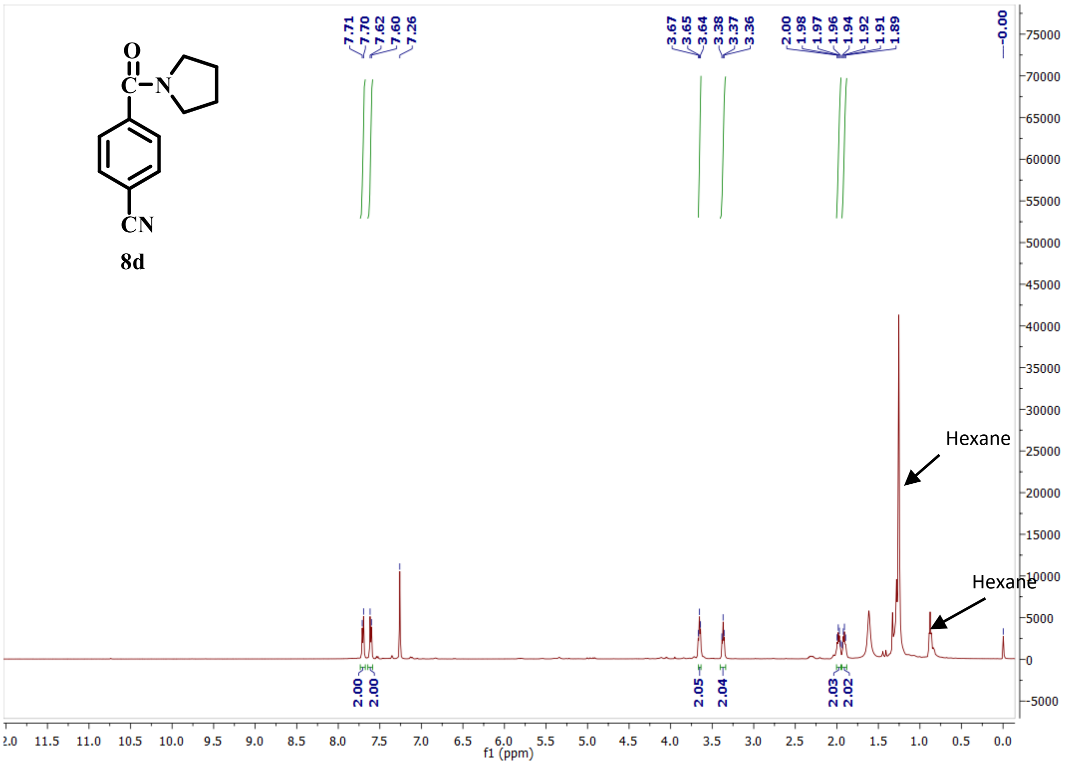

Figure S47. ^{1}H NMR Spectra ($CDCl_3$, 500 MHz, ppm) of compound **8d.**

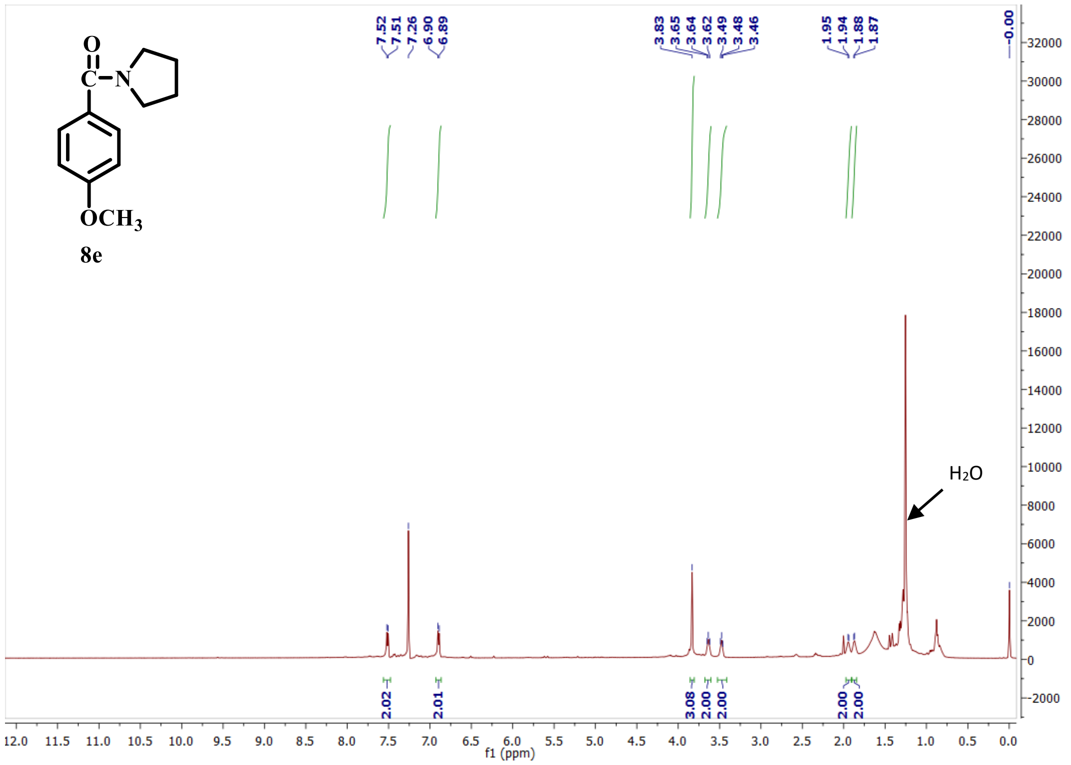

Figure S48. ^{1}H NMR Spectra ($CDCl_3$, 500 MHz, ppm) of compound **8e.**

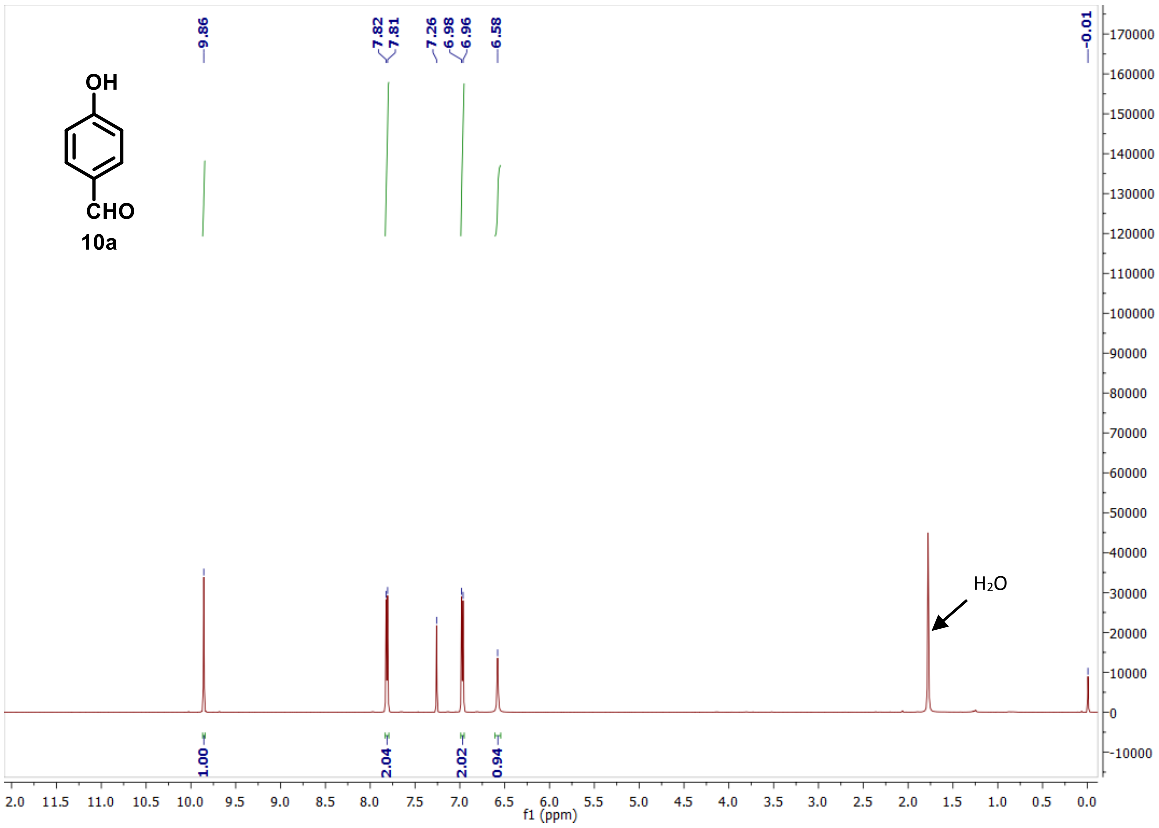

Figure S49. ^{1}H NMR Spectra ($CDCl_3$, 500 MHz, ppm) of compound **10a.**

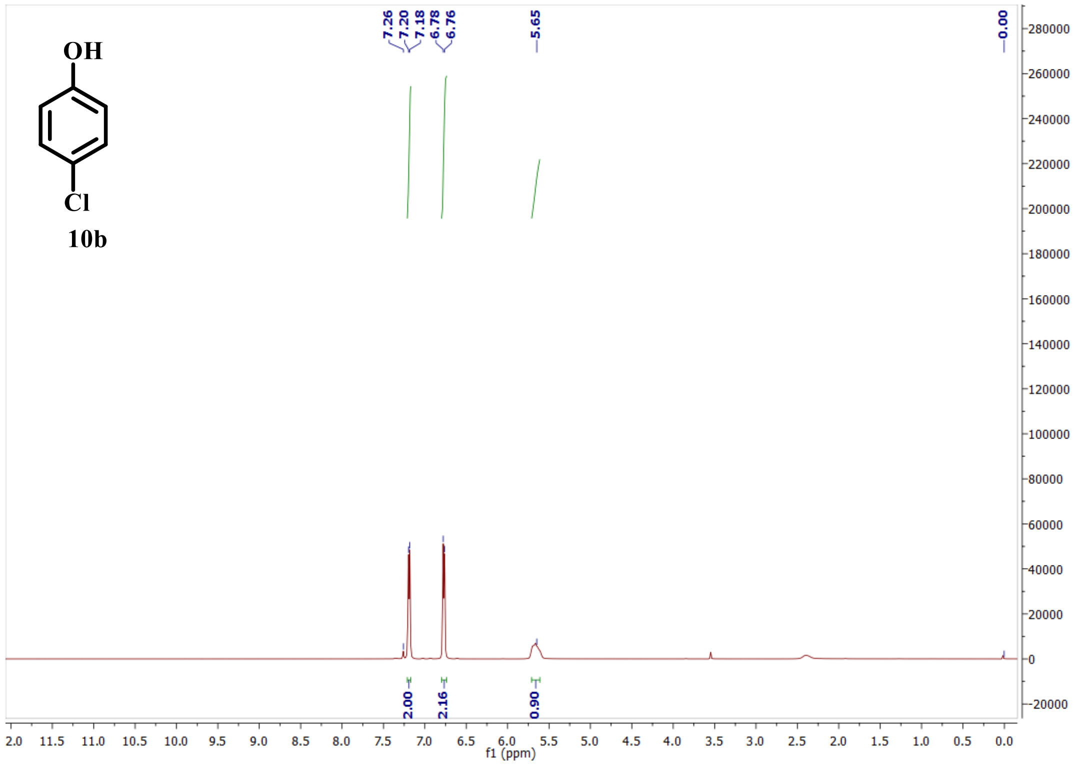

Figure S50. ^{1}H NMR Spectra ($CDCl_3$, 500 MHz, ppm) of compound **10b.**

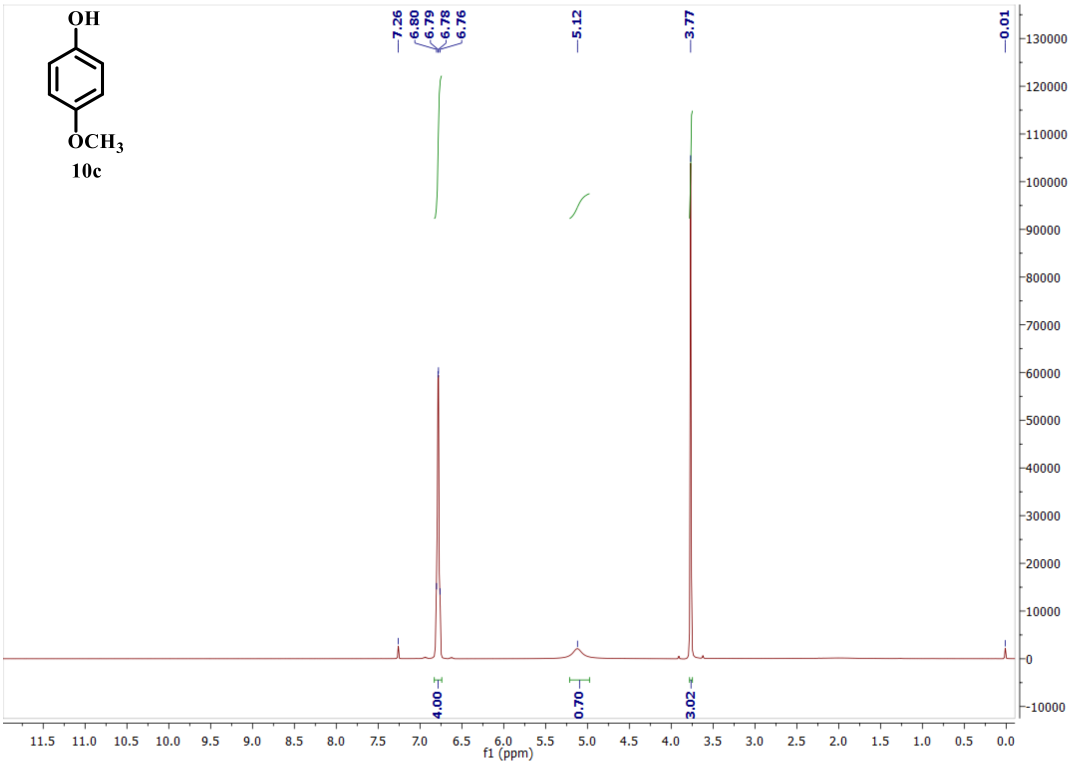

Figure S51. ^{1}H NMR Spectra ($CDCl_3$, 500 MHz, ppm) of compound **10c.**

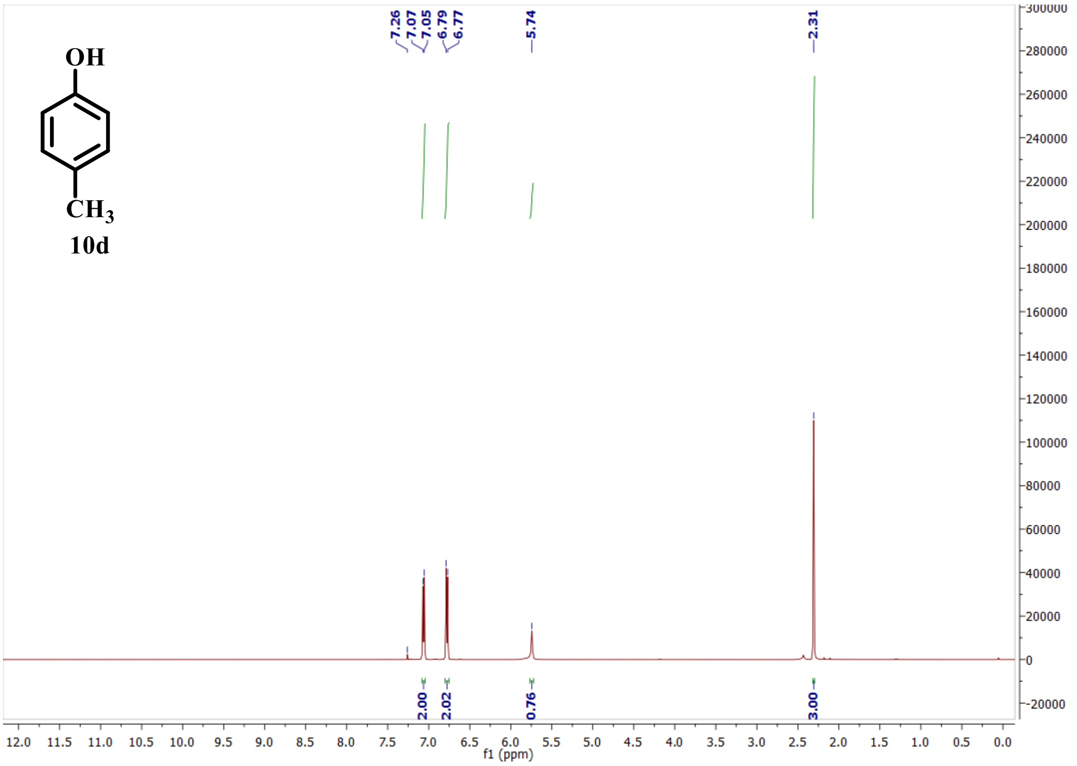

Figure S52. ^{1}H NMR Spectra ($CDCl_3$, 500 MHz, ppm) of compound **10d.**

Experimental details of density functional theory:

All calculations were performed using density functional theory (DFT) and (TDFT) with GAUSSIAN 09 suite programs. We carried out density functional theory (DFT) calculation sets in the Gaussian 09 program. All the structures were completely optimized using a combined basis set: using B3LYP functional and 6-31G (d,p) basis set. The frequency calculations were performed at the same level at 298 K and 1 atm, and vibrational analysis was performed to confirm the optimized stationary points as true minima with no imaginary frequency.

DIPY

Center	Atomic	Forces (Hartrees/Bohr)		
Number	Number	X	Y	Z
1	6	-0.000034402	0.000006688	-0.000001330
2	6	0.000001072	0.000007024	-0.000003836
3	6	-0.000006816	-0.000021808	0.000008669
4	6	-0.000085639	-0.000034795	-0.000143305
5	6	0.000405802	-0.000044025	0.000369389
6	6	0.000136914	0.000613205	-0.000080279
7	1	-0.000005049	-0.000000432	0.000009145
8	1	0.000000406	-0.000000950	-0.000000434
9	1	-0.000187211	0.000002533	-0.000129303
10	1	-0.000236775	-0.000541055	-0.000050817
11	6	-0.000003043	0.000018995	0.000011412
12	6	0.000000888	-0.000009433	-0.000002596
13	6	-0.000003237	0.000004211	0.000001849
14	6	0.000003043	-0.000004635	-0.000003024
15	6	0.000002236	-0.000003276	0.000001067
16	6	-0.000003658	0.000002842	-0.000001680
17	1	-0.000000075	0.000000675	0.000000999
18	6	-0.000000344	0.000004154	0.000002660
19	1	0.000000188	0.000000301	-0.000000815
20	6	0.000001713	-0.000004328	0.000000092
21	1	0.000000414	0.000000351	0.000000846
22	1	-0.000000515	-0.000000365	-0.000000470
23	6	0.000006370	0.000005989	-0.000000916
24	6	-0.000003500	-0.000003939	0.000001210
25	6	-0.000002500	-0.000000443	0.000001480
26	6	0.000004199	0.000001649	0.000000132
27	1	0.000000362	0.000000868	0.000000207
28	6	0.000000248	0.000000631	0.000000766
29	1	0.000000155	0.000000271	0.000000704
30	6	-0.000001916	0.000000071	-0.000000935
31	1	-0.000001098	-0.000000443	-0.000000690
32	1	0.000000116	-0.000001430	0.000000272
33	6	-0.000001462	0.000002824	-0.000000836
34	6	0.000001528	-0.000000847	0.000001483

35	6	0.000000247	-0.000001665	-0.000001601
36	6	-0.000000561	0.000000601	0.000000657
37	1	-0.000000020	-0.000000069	-0.000000660
38	6	-0.000000408	0.000000872	-0.000000894
39	1	-0.000000017	0.000000288	0.000000555
40	6	-0.000000072	0.000000355	-0.000000100
41	1	0.000000069	-0.000000510	-0.000000605
42	1	0.000000279	-0.000000224	0.000000233
43	6	0.000000046	-0.000000925	-0.000000600
44	6	-0.000000663	0.000000876	-0.000000084
45	6	0.000000455	-0.000000433	-0.000000142
46	6	-0.000001136	0.000000339	0.000003091
47	1	0.000000278	-0.000000083	-0.000001130
48	6	0.000001043	0.000003319	-0.000002758
49	1	-0.000000251	-0.000000100	0.000000379
50	6	0.000000719	-0.000001918	0.000001728
51	6	-0.000000113	-0.000000090	-0.000000941
52	6	0.000000187	0.000000066	-0.000000356
53	1	-0.000000006	0.000000145	-0.000000550
54	6	0.000001294	0.000000597	-0.000000087
55	6	-0.000000441	-0.000000154	-0.000000639
56	1	0.000000301	-0.000000242	-0.000000554
57	1	-0.000000360	-0.000000025	-0.000000512
58	1	0.000000017	-0.000000175	-0.000000483
59	6	0.000001256	-0.000002453	-0.000002317
60	6	-0.000000171	-0.000000097	-0.000000555
61	6	-0.000001555	-0.000002153	-0.000001922
62	6	-0.000000262	0.000000668	-0.000000623
63	1	-0.000000113	0.000000487	0.000000117
64	6	0.000000548	0.000000503	0.000000569
65	1	0.000000184	0.000000508	-0.000000503
66	1	-0.000000069	-0.000000111	-0.000000204
67	1	-0.000000018	0.000000172	-0.000000527
68	7	-0.000000107	-0.000001454	-0.000001205
69	7	0.000000049	0.000003156	0.000000926
70	7	-0.000001288	0.000000480	-0.000001400
71	7	0.000022693	-0.000005439	0.000012898
72	7	-0.000010283	0.000005171	0.000002905
73	6	0.000000746	0.000000924	0.000000508
74	6	-0.000000623	-0.000001378	-0.000000423
75	6	-0.000001151	-0.000001138	0.000001157
76	6	0.000000529	0.000000518	0.000001254
77	1	0.000000411	0.000000823	0.000000770
78	6	0.000000082	0.000000537	-0.000000809
79	1	0.000000305	0.000000506	-0.000000507
80	6	-0.000001747	-0.000002723	-0.000000083
81	1	0.000000017	-0.000000139	0.000000298
82	1	-0.000000229	0.000000023	0.000000130
83	6	0.000002641	0.000003742	-0.000000659
84	6	-0.000001775	-0.000000797	0.000001010
85	6	-0.000000848	-0.000001626	0.000000421
86	6	0.000000661	0.000001315	-0.000002270
87	1	0.000000156	-0.000000186	0.000000433
88	6	0.000002240	0.000001419	-0.000000862
89	1	-0.000000255	-0.000000426	0.000000323
90	6	-0.000002095	0.000001020	0.000000646
91	6	0.000000052	-0.000000673	0.000001485
92	6	0.000000918	0.000000010	-0.000000027
93	1	0.000000070	-0.000000265	-0.000000283
94	6	-0.000000443	-0.000002256	0.000002363

95	6	-0.000000112	0.000000397	-0.000000696
96	1	-0.000000071	-0.000000281	0.000000191
97	1	0.000000248	0.000000594	0.000000134
98	1	0.000000109	-0.000000140	0.000000233
99	6	0.000000084	-0.000000315	0.000002432
100	6	0.000000182	-0.000000365	-0.000000908
101	6	-0.000000022	0.000001269	0.000000393
102	1	-0.000000369	-0.000000082	0.000000048
103	6	0.000000241	-0.000000167	-0.000000998
104	6	-0.000000308	-0.000000448	0.000000828
105	1	-0.000000113	-0.000000045	0.000000214
106	1	-0.000000221	0.000000278	0.000000131
107	1	-0.000000130	0.000000152	-0.000000016
108	7	-0.000000929	-0.000002331	0.000001437
109	7	-0.000000431	0.000000435	-0.000000359
110	7	0.000002014	0.000000463	-0.000001164

TETPY

Center Number	Atomic Number	Atomic Type	Coordinates (Angstroms) X	Y	Z
1	6	0	-1.634045	0.713650	-0.257268
2	6	0	-1.653367	-0.643413	0.238470
3	7	0	-0.506631	-1.344854	0.396545
4	6	0	0.656519	-0.694591	0.135415
5	6	0	0.675733	0.705354	-0.133088
6	7	0	-0.467631	1.385317	-0.403211
7	6	0	1.872030	1.555027	-0.056232
8	6	0	1.829330	-1.576473	0.068557
9	6	0	1.919331	2.756597	-0.803705
10	6	0	3.002485	3.611205	-0.716665
11	6	0	4.096470	3.332434	0.136087
12	6	0	4.034820	2.148282	0.905182
13	6	0	2.952709	1.290196	0.820955
14	6	0	2.923111	-1.344196	-0.802148
15	6	0	3.980943	-2.232439	-0.877266
16	6	0	4.004953	-3.415983	-0.104249
17	6	0	2.897805	-3.661323	0.742408
18	6	0	1.838216	-2.777237	0.819432
19	6	0	5.242327	4.248485	0.234917
20	6	0	5.124813	-4.363802	-0.192454
21	6	0	6.534245	3.775956	0.547982
22	6	0	7.616697	4.639532	0.637797
23	6	0	7.463180	6.022678	0.431222
24	6	0	6.175860	6.499549	0.123607
25	6	0	5.094942	5.635423	0.022601
26	6	0	6.429368	-3.931258	-0.512539
27	6	0	7.487452	-4.825235	-0.591626
28	6	0	7.296499	-6.200988	-0.366435
29	6	0	5.996681	-6.638130	-0.051723
30	6	0	4.940030	-5.743693	0.038046
31	6	0	8.616434	6.944908	0.535627
32	6	0	8.423693	-7.155871	-0.458461
33	6	0	9.897257	6.567514	0.110925
34	6	0	10.960057	7.473242	0.225009
35	7	0	10.814129	8.702641	0.737519

36	6	0	9.594921	9.074166	1.151036
37	6	0	8.476390	8.234697	1.064662
38	6	0	9.716044	-6.806270	-0.044733
39	6	0	10.753084	-7.742848	-0.145684
40	7	0	10.571576	-8.976999	-0.634927
41	6	0	9.341069	-9.322339	-1.037571
42	6	0	8.246486	-8.450798	-0.963486
43	6	0	9.465024	10.451233	1.718824
44	6	0	12.332399	7.090099	-0.228422
45	6	0	9.170668	-10.705968	-1.578015
46	6	0	12.136987	-7.389359	0.296531
47	7	0	9.865408	-11.682623	-0.971534
48	6	0	9.726053	-12.926801	-1.434675
49	6	0	8.904681	-13.271454	-2.510573
50	6	0	8.189645	-12.255447	-3.140167
51	6	0	8.322297	-10.951734	-2.668266
52	6	0	12.347250	-6.615164	1.447658
53	6	0	13.652156	-6.308046	1.825748
54	6	0	14.704395	-6.780180	1.044869
55	6	0	14.394029	-7.545354	-0.081744
56	7	0	13.149469	-7.849882	-0.456524
57	6	0	12.517862	6.328352	-1.392078
58	6	0	13.812746	5.992609	-1.780124
59	6	0	14.880073	6.424786	-0.996556
60	6	0	14.594259	7.180730	0.142684
61	7	0	13.359625	7.512171	0.527241
62	7	0	10.184943	11.419874	1.129039
63	6	0	10.082696	12.657905	1.617636
64	6	0	9.275427	13.003981	2.703699
65	6	0	8.534012	11.996187	3.315746
66	6	0	8.627812	10.699024	2.817224
67	6	0	-2.862843	1.352326	-0.516863
68	6	0	-4.098261	0.738333	-0.273913
69	6	0	-4.116791	-0.608321	0.232213
70	6	0	-2.900551	-1.252577	0.487097
71	6	0	-5.320481	1.545577	-0.479724
72	6	0	-5.360125	-1.387983	0.426046
73	6	0	-6.363982	1.584410	0.468445
74	6	0	-7.478298	2.390252	0.280702
75	6	0	-7.610009	3.199098	-0.862826
76	6	0	-6.567368	3.169757	-1.806651
77	6	0	-5.449258	2.367964	-1.617548
78	6	0	-6.380795	-1.425428	-0.545708
79	6	0	-7.510657	-2.213932	-0.372632
80	6	0	-7.684066	-2.995352	0.783815
81	6	0	-6.667356	-2.961842	1.754873
82	6	0	-5.529209	-2.185895	1.575175
83	6	0	-8.806193	4.047433	-1.064420
84	6	0	-8.893986	-3.827633	0.972226
85	6	0	-10.067430	3.648022	-0.606163
86	6	0	-11.169248	4.487663	-0.806680
87	7	0	-11.079877	5.669075	-1.434066
88	6	0	-9.880975	6.061669	-1.885366
89	6	0	-8.725560	5.288215	-1.716361
90	6	0	-9.521621	-4.467183	-0.105216
91	6	0	-10.662029	-5.249367	0.118931
92	7	0	-11.202319	-5.415714	1.334031
93	6	0	-10.614692	-4.805183	2.372142
94	6	0	-9.467036	-4.013219	2.237593
95	6	0	-11.337192	-5.944275	-1.019998

96	6	0	-11.241541	-5.003844	3.715060
97	6	0	-12.523697	4.088902	-0.327094
98	6	0	-9.823786	7.385718	-2.576433
99	6	0	-13.619675	4.947643	-0.496261
100	6	0	-14.865575	4.536818	-0.033523
101	6	0	-14.982804	3.289373	0.579772
102	6	0	-13.833693	2.506949	0.703237
103	7	0	-12.630251	2.886584	0.266210
104	7	0	-10.680145	8.331374	-2.152141
105	6	0	-10.645199	9.522571	-2.753517
106	6	0	-9.775579	9.845711	-3.797297
107	6	0	-8.894949	8.862034	-4.241370
108	6	0	-8.918483	7.613810	-3.624898
109	6	0	-10.586495	-6.514184	-2.060005
110	6	0	-11.252264	-7.151233	-3.104153
111	6	0	-12.644076	-7.200626	-3.080092
112	6	0	-13.302506	-6.607876	-2.000405
113	7	0	-12.679460	-5.994520	-0.991725
114	7	0	-11.739489	-6.223276	3.979875
115	6	0	-12.301393	-6.421957	5.174370
116	6	0	-12.403509	-5.440164	6.162433
117	6	0	-11.890442	-4.175630	5.883199
118	6	0	-11.299960	-3.951590	4.642030
119	1	0	1.083477	2.983315	-1.454387
120	1	0	3.025136	4.498810	-1.340890
121	1	0	4.820768	1.934729	1.622353
122	1	0	2.910845	0.423231	1.469740
123	1	0	2.910015	-0.477739	-1.452859
124	1	0	4.776791	-2.042854	-1.590174
125	1	0	2.891403	-4.546591	1.370187
126	1	0	0.992113	-2.978811	1.465070
127	1	0	6.693062	2.713180	0.698515
128	1	0	8.594251	4.242029	0.892470
129	1	0	6.026929	7.559530	-0.057036
130	1	0	4.113707	6.042265	-0.197865
131	1	0	6.617695	-2.875436	-0.676910
132	1	0	8.475216	-4.458050	-0.852188
133	1	0	5.818796	-7.690959	0.143645
134	1	0	3.948503	-6.121175	0.264185
135	1	0	10.065869	5.582899	-0.310817
136	1	0	7.509368	8.580505	1.412501
137	1	0	9.913426	-5.819451	0.358969
138	1	0	7.269299	-8.776439	-1.302229
139	1	0	10.300420	-13.693304	-0.916894
140	1	0	8.835203	-14.302533	-2.841995
141	1	0	7.547030	-12.470228	-3.989028
142	1	0	7.799476	-10.133248	-3.151973
143	1	0	11.505764	-6.284891	2.047822
144	1	0	13.841823	-5.719616	2.718770
145	1	0	15.738055	-6.568644	1.299068
146	1	0	15.189261	-7.931888	-0.717129
147	1	0	11.665688	6.029787	-1.993753
148	1	0	13.983423	5.413036	-2.682725
149	1	0	15.906804	6.189852	-1.258190
150	1	0	15.401808	7.536510	0.780511
151	1	0	10.676733	13.418107	1.112906
152	1	0	9.236600	14.029709	3.056085
153	1	0	7.900844	12.211914	4.171443
154	1	0	8.083649	9.886111	3.286567
155	1	0	-2.823958	2.387182	-0.839251

156	1	0	-2.890223	-2.287513	0.811594
157	1	0	-6.280631	0.995210	1.374614
158	1	0	-8.246419	2.419943	1.046944
159	1	0	-6.651921	3.755982	-2.716676
160	1	0	-4.672280	2.345564	-2.375284
161	1	0	-6.275137	-0.837505	-1.450522
162	1	0	-8.283349	-2.206953	-1.135272
163	1	0	-6.760164	-3.571050	2.648901
164	1	0	-4.759475	-2.178700	2.340573
165	1	0	-10.223165	2.695476	-0.115776
166	1	0	-7.764003	5.670019	-2.040419
167	1	0	-9.123622	-4.362230	-1.108248
168	1	0	-9.028023	-3.540582	3.109117
169	1	0	-13.465037	5.905007	-0.978957
170	1	0	-15.733001	5.180419	-0.149397
171	1	0	-15.935472	2.928293	0.953786
172	1	0	-13.883549	1.527398	1.175749
173	1	0	-11.349959	10.264022	-2.379919
174	1	0	-9.796002	10.833627	-4.246118
175	1	0	-8.208003	9.057928	-5.059601
176	1	0	-8.264047	6.819036	-3.966304
177	1	0	-9.502324	-6.480492	-2.035530
178	1	0	-10.692681	-7.608029	-3.915360
179	1	0	-13.208021	-7.686483	-3.869946
180	1	0	-14.389961	-6.625714	-1.946004
181	1	0	-12.690655	-7.422766	5.355236
182	1	0	-12.874038	-5.663632	7.114604
183	1	0	-11.956207	-3.374125	6.613345
184	1	0	-10.913804	-2.971345	4.382974

CNDIPY

Center Number	Atomic Number	Atomic Type	Coordinates (Angstroms) X	Y	Z
1	6	0	-0.687163	8.510294	-0.366840
2	6	0	0.494038	8.500863	0.467264
3	7	0	1.135412	7.390499	0.750113
4	6	0	0.659589	6.209194	0.249757
5	6	0	-0.705195	6.208643	-0.249713
6	7	0	-1.314822	7.330683	-0.593803
7	6	0	-1.582083	5.016924	-0.263817
8	6	0	1.569084	5.086650	0.224399
9	6	0	-1.421602	3.951896	0.658741
10	6	0	-2.309879	2.894229	0.682542
11	6	0	-3.391393	2.819558	-0.229875
12	6	0	-3.540009	3.888565	-1.159000
13	6	0	-2.674575	4.961296	-1.161616
14	6	0	2.710771	5.063232	1.070157
15	6	0	3.588080	3.996913	1.058735
16	6	0	3.410916	2.891429	0.189929
17	6	0	2.298754	2.931957	-0.676469
18	6	0	1.408201	3.992505	-0.668717
19	6	0	-4.328157	1.703746	-0.212529
20	6	0	4.360891	1.768318	0.175598
21	6	0	-5.086676	1.356652	-1.361915
22	6	0	-5.973690	0.298311	-1.345510
23	6	0	-6.178383	-0.467591	-0.175282
24	6	0	-5.431069	-0.123621	0.974772

25	6	0	-4.527751	0.920472	0.955341
26	6	0	4.591685	1.010275	-0.992020
27	6	0	5.493920	-0.044127	-1.004675
28	6	0	6.212288	-0.405070	0.150029
29	6	0	5.981375	0.342915	1.318940
30	6	0	5.084828	1.402327	1.330398
31	6	0	-7.141102	-1.583995	-0.154336
32	6	0	7.178620	-1.525186	0.137451
33	6	0	-7.430568	-2.315608	-1.315879
34	6	0	-8.353369	-3.365185	-1.252292
35	7	0	-8.985738	-3.701218	-0.116943
36	6	0	-8.718411	-3.007559	1.000622
37	6	0	-7.804006	-1.948917	1.027029
38	6	0	7.933656	-1.828201	-1.006890
39	6	0	8.835770	-2.898484	-0.982772
40	7	0	9.009742	-3.667353	0.100623
41	6	0	8.295486	-3.387703	1.200663
42	6	0	7.378189	-2.332600	1.264378
43	6	0	-9.443520	-3.399628	2.243434
44	6	0	-8.665545	-4.160610	-2.474574
45	6	0	8.520780	-4.270165	2.381484
46	6	0	9.659694	-3.232177	-2.184450
47	7	0	7.838646	-3.975651	3.502674
48	6	0	8.026192	-4.752111	4.572905
49	6	0	8.888966	-5.848876	4.591201
50	6	0	9.592431	-6.150602	3.424932
51	6	0	9.408785	-5.352969	2.300270
52	6	0	10.132419	-2.223009	-3.038040
53	6	0	10.894246	-2.576976	-4.148580
54	6	0	11.163660	-3.924805	-4.374863
55	6	0	10.656863	-4.855479	-3.465386
56	7	0	9.925982	-4.532850	-2.395747
57	6	0	-9.579235	-5.222057	-2.434315
58	6	0	-9.833002	-5.930376	-3.605203
59	6	0	-9.171123	-5.561549	-4.775366
60	6	0	-8.277957	-4.490167	-4.716175
61	7	0	-8.024893	-3.801158	-3.601238
62	7	0	-9.143367	-2.704515	3.355081
63	6	0	-9.773728	-3.025021	4.487252
64	6	0	-10.726203	-4.041618	4.578931
65	6	0	-11.034092	-4.759420	3.424210
66	6	0	-10.385537	-4.436770	2.235789
67	6	0	-1.290628	9.704435	-0.833750
68	7	0	-1.767810	10.690422	-1.241502
69	6	0	0.984328	9.716847	1.062399
70	7	0	1.371896	10.705900	1.536771
71	1	0	-0.600389	3.978551	1.363372
72	1	0	-2.146201	2.092813	1.393984
73	1	0	-4.376447	3.889747	-1.848880
74	1	0	-2.822102	5.792315	-1.840418
75	1	0	2.879700	5.905729	1.730688
76	1	0	4.454644	4.027321	1.711974
77	1	0	2.126504	2.111191	-1.365927
78	1	0	0.597571	3.999904	-1.389034
79	1	0	-4.949711	1.910438	-2.283212
80	1	0	-6.538550	0.067166	-2.241697
81	1	0	-5.554645	-0.700054	1.884724
82	1	0	-3.988340	1.162872	1.863239
83	1	0	4.075574	1.270379	-1.910462
84	1	0	5.632298	-0.609568	-1.921226

85	1	0	6.526521	0.100513	2.225835
86	1	0	4.924038	1.943761	2.256960
87	1	0	-6.944095	-2.119767	-2.262568
88	1	0	-7.660984	-1.420954	1.960799
89	1	0	7.822485	-1.233191	-1.906177
90	1	0	6.821486	-2.184120	2.180934
91	1	0	7.458548	-4.484664	5.462714
92	1	0	9.001757	-6.443895	5.491814
93	1	0	10.273539	-6.996202	3.392110
94	1	0	9.923618	-5.538442	1.365443
95	1	0	9.928738	-1.180112	-2.819839
96	1	0	11.277775	-1.812370	-4.817895
97	1	0	11.753138	-4.250476	-5.225894
98	1	0	10.848061	-5.918201	-3.606945
99	1	0	-10.065665	-5.468818	-1.499262
100	1	0	-10.536237	-6.757914	-3.603867
101	1	0	-9.338034	-6.086014	-5.710372
102	1	0	-7.741112	-4.171566	-5.607652
103	1	0	-9.505554	-2.441424	5.365836
104	1	0	-11.207731	-4.258768	5.526620
105	1	0	-11.769272	-5.558357	3.448214
106	1	0	-10.589391	-4.962414	1.311647

References:

(1) Lin, M.; Wang, Z.; Fang, H.; Liu, L.; Yin, H.; Yan, C.- H.; Fu, X. Metal-free Aerobic Oxidative Coupling of Amines in Dimethyl Sulfoxide *via* a Radical Pathway. *RSC Adv.*, **2016**, 6, 1086.

(2) Deol, H.; Kumar, M.; Bhalla, V. Exploring Organic Photosensitizers Based on Hemicyanine Derivatives: A Sustainable Approach for Preparation of Amide Linkages. *RSC Adv*. **2018**, *8*, 31237-31245.

(3) Kumar, I.; Sharma, R.; Kumar, R.; Kumar, R.; Sharma, U. C70 Fullerene-Catalyzed Metal-Free Photocatalytic ipso- Hydroxylation of Aryl Boronic Acids: Synthesis of Phenols. *Adv. Synth. Catal*. **2018**, *360*, 1–8.

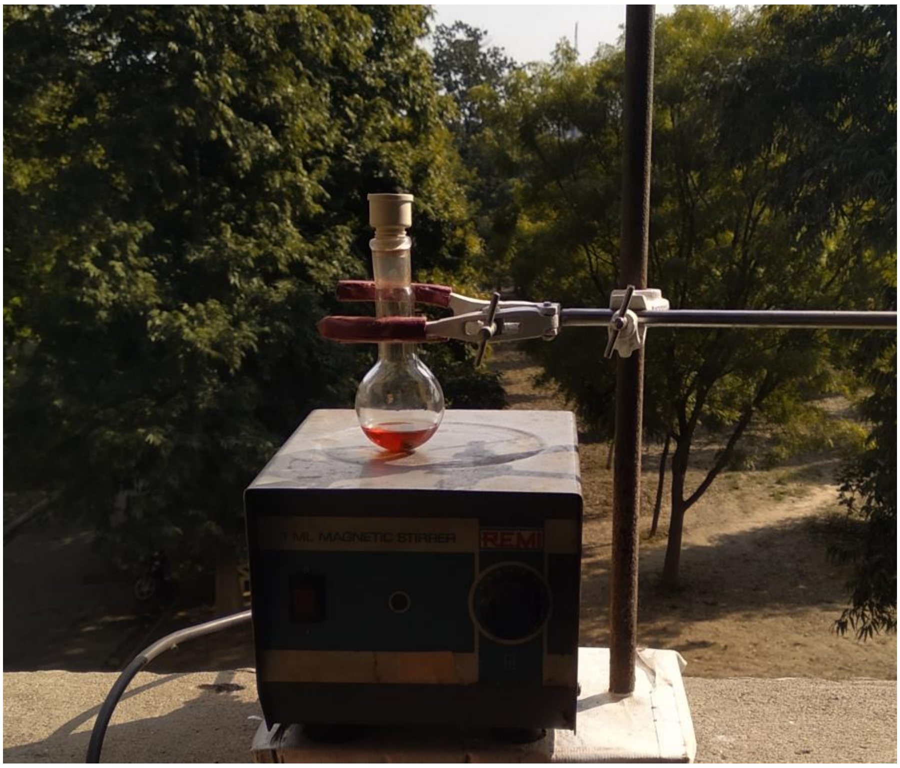

Photocatalytic reaction setup for the oxidative amidation of aromatic aldehydes under aerial conditions with irradiation of natural sunlight.
